# Long-Term Relationships between Synaptic Tenacity, Synaptic Remodeling, and Network Activity

**DOI:** 10.1371/journal.pbio.1000136

**Published:** 2009-06-23

**Authors:** Amir Minerbi, Roni Kahana, Larissa Goldfeld, Maya Kaufman, Shimon Marom, Noam E. Ziv

**Affiliations:** 1 Department of Physiology and Biophysics, Technion Faculty of Medicine, Haifa, Israel; 2 Network Biology Research Laboratories, Lorry Lokey Interdisciplinary Center for Life Sciences and Engineering, Haifa, Israel; 3 The Rappaport Family Institute for Research in the Medical Sciences, Haifa, Israel; Salk Institute for Biological Studies, United States of America

## Abstract

Synaptic plasticity is widely believed to constitute a key mechanism for modifying functional properties of neuronal networks. This belief implicitly implies, however, that synapses, when not driven to change their characteristics by physiologically relevant stimuli, will maintain these characteristics over time. How tenacious are synapses over behaviorally relevant time scales? To begin to address this question, we developed a system for continuously imaging the structural dynamics of individual synapses over many days, while recording network activity in the same preparations. We found that in spontaneously active networks, distributions of synaptic sizes were generally stable over days. Following individual synapses revealed, however, that the apparently static distributions were actually steady states of synapses exhibiting continual and extensive remodeling. In active networks, large synapses tended to grow smaller, whereas small synapses tended to grow larger, mainly during periods of particularly synchronous activity. Suppression of network activity only mildly affected the magnitude of synaptic remodeling, but dependence on synaptic size was lost, leading to the broadening of synaptic size distributions and increases in mean synaptic size. From the perspective of individual neurons, activity drove changes in the relative sizes of their excitatory inputs, but such changes continued, albeit at lower rates, even when network activity was blocked. Our findings show that activity strongly drives synaptic remodeling, but they also show that significant remodeling occurs spontaneously. Whereas such spontaneous remodeling provides an explanation for “synaptic homeostasis” like processes, it also raises significant questions concerning the reliability of individual synapses as sites for persistently modifying network function.

## Abstract 2

Long term time-lapse imaging reveals that individual synapses undergo significant structural remodeling not only when driven by activity, but also when network activity is absent, raising questions about how reliably individual synapses maintain connections.

## Author Summary

Neurons communicate via synapses, and it is believed that activity-dependent modifications to synaptic connections—synaptic plasticity—is a fundamental mechanism for stably altering the function of neuronal networks. This belief implies that synapses, when *not* driven to change their properties by physiologically relevant stimuli, should preserve their individual properties over time. Otherwise, physiologically relevant modifications to network function would be gradually lost or become inseparable from stochastically occurring changes in the network. So do synapses actually preserve their properties over behaviorally relevant time scales? To begin to address this question, we examined the structural dynamics of individual postsynaptic densities for several days, while recording and manipulating network activity levels in the same networks. We found that as expected in highly active networks, individual synapses undergo continual and extensive remodeling over time scales of many hours to days. However, we also observed, that synaptic remodeling continues at very significant rates even when network activity is completely blocked. Our findings thus indicate that the capacity of synapses to preserve their specific properties might be more limited than previously thought, raising intriguing questions about the long-term reliability of individual synapses.

## Introduction

Synapses are widely believed to constitute key loci for modifying the functional properties of neuronal networks, possibly providing the basis for phenomena collectively referred to as learning and memory [1],[2]. Indeed, an overwhelming body of literature supports the notion that synapses are “plastic”, that is, change their functional characteristics in response to specific activation patterns. The hypothesis that activity-dependent changes to synaptic characteristics constitutes a key mechanism for modifying neuronal network function also implies, however, that synapses, when *not* driven to change their characteristics by physiologically relevant stimuli, should retain these characteristics over time. Otherwise, physiologically relevant modifications to network function would be gradually lost due to stochastic, spurious changes or spontaneous drift. Thus, it might be expected that the capacity of synapses for directed change—synaptic plasticity—should be accompanied by a tendency to retain their characteristics at all other times, a phenomenon we will refer to here as “synaptic tenacity”.

The advent of molecular imaging techniques and the ability to study the molecular dynamics of specific molecules are gradually leading to the realization that synapses are not static, rigid structures; rather, they are made of multimolecular protein ensembles that exhibit significant dynamics at time scales of seconds to hours. Such dynamics include the recruitment and dispersal of regulatory constituents, lateral diffusion, endocytosis and exocytosis of postsynaptic neurotransmitter receptors, cytoskeletal dynamics and spine “morphing”, loss, incorporation, and turnover of scaffold molecules, and the interchange of synaptic molecules, multimolecular complexes, and synaptic vesicles among neighboring synapses (reviewed in [3]–[11]). When considering the bewildering dynamics exhibited by synaptic molecules, it becomes apparent that the long-term tenacity of synaptic structure and, by extension, synaptic function is not at all an obvious outcome. Yet to date, very little is known on the long-term tenacity of individual synapses [12].

Despite the molecular dynamics of synaptic constituents, most central nervous system (CNS) synapses appear to be quite persistent, although some degree of synapse formation and elimination is observed, depending on brain region, type of synapse, animal age, and imaging techniques [13]–[20] (reviewed in [8],[21]). Interestingly, however, even persistent synapses, when examined over long time scales (days), seem to exhibit considerable morphological changes (for example, [14],[15],[17],[22],[23]; see also [18]). In most of these studies, it was surmised that the observed changes in synaptic morphology represented structural manifestations of synaptic plasticity processes.

In most of the aforementioned studies, synapses were visualized by means of volume-filling fluorescent dyes (mainly enhanced green fluorescent protein [EGFP] or its spectral variants) and identified on the basis of typical pre- and postsynaptic morphological features (i.e., axonal varicosities and dendritic spines, respectively), whereas functionally relevant reporters, such as synaptic vesicle, postsynaptic receptor, active zone, or postsynaptic density (PSD) molecules were rarely used. Furthermore, even though manipulations aimed at altering network activity were performed in some of these studies, actual network activity was not recorded. Thus, the actual relationships between synaptic tenacity, synaptic remodeling, and network activity over these long time scales remained unknown.

To evaluate the tenacity of individual synaptic structures over behaviorally relevant time scales and differentiate between activity-dependent and activity independent-synaptic remodeling, an experimental system is needed in which both structural dynamics of individual synapses and electrical activity can be monitored continuously and simultaneously at sufficiently high temporal resolutions for very long periods. At present, this is an extremely challenging requirement, in particular where in vivo studies are concerned. We therefore developed a novel system, based on networks of rat cortical neurons in primary culture, that allowed us to continuously follow and record the structural dynamics of individual PSDs over time scales of minutes to weeks while concomitantly recording (and manipulating) network activity in the same preparations. We find that the vast majority of PSDs in this preparation undergo significant, continuous remodeling over time scales of many hours and days. The direction and extent of PSD remodeling are strongly affected by network activity levels, but remodeling does not cease upon suppression or elimination of activity. Our findings, described below, thus indicate that the tenacity exhibited by individual synapses over time scales of days is rather limited and may indicate that structural (and by extension, functional) properties of individual synapses experience significant drift over long durations.

## Results

### Long-Term Time-Lapse Imaging of Postsynaptic Structures with Concomitant Recording of Network Activity

**Figure 1:**
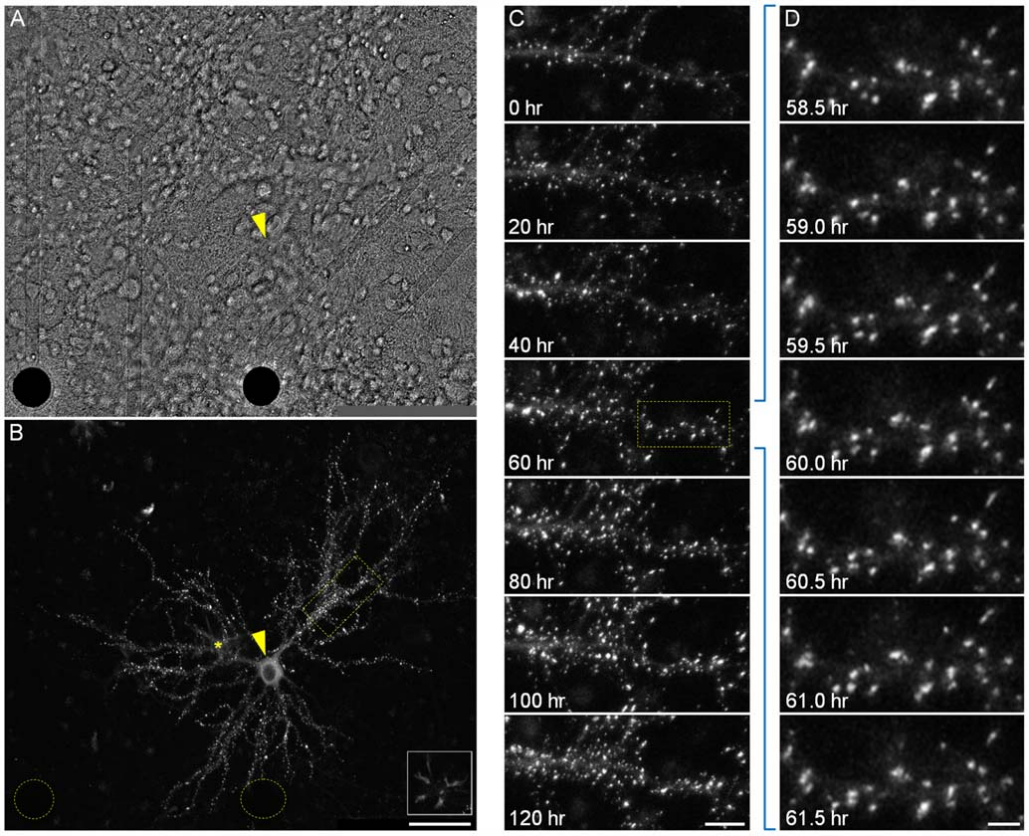
Long-term imaging of postsynaptic sites. (A) Rat cortical neurons growing on a thin-glass MEA dish. Two electrodes (opaque circles at the bottom of the image) and their transparent leads are visible. The yellow arrowhead indicates the neuron shown in (B). (B) A single neuron expressing PSD-95:GFP (yellow arrowhead) in the same field of view as (A). Fluorescent puncta represent postsynaptic sites formed on dendritic spines and shafts. On rare occasions, other cell types (a microglia cell in this image) also expressed the fluorescent protein (asterisk and inset). The positions of the two electrodes shown in (A) are denoted as yellow dotted-line circles. (C) A 5-d time-lapse series (30-min intervals, or 48 images/day) of the region enclosed in a yellow rectangle in (B) (only a small subset of the data is shown here). (D) Magnification of region enclosed in yellow rectangle in (C), demonstrating the actual temporal and spatial resolution of imaging data collected in these experiments. All images in (B–D) are maximal intensity projections of 14 images collected at 14 focal planes spaced 0.8 µm apart. Inset in (B) includes only the two bottommost sections. Bars in (A and B) indicate 50 µm; in (C), 10 µm; and in (D), 3 µm.

In order to examine the tenacity of individual synapses, a system was needed that would allow us to record the structural dynamics of individual synaptic structures while concomitantly recording network activity in the same preparations and to do so continuously for many days. The experimental system we developed for this purpose was based on primary cultures of cortical neurons obtained from neonatal rats and plated on substrate-integrated multielectrode array (MEA) dishes [24]–[27]. To allow for the use of high numerical aperture oil-immersion objectives (that typically have short working distances), we used special MEA dishes made of very thin glass (180 µm) that are ideally suited for high-resolution imaging. Each dish contained 59 electrodes arranged in an 8×8 grid with interelectrode distances of 200 µm. Although the flat 30-µm diameter electrodes were opaque, the leads were transparent, resulting in minimal optical obstructions in the imaged regions (Figure 1A).

PSDs were visualized by expressing an EGFP-tagged variant of the PSD molecule PSD-95 (PSD-95:GFP). PSD-95 [28],[29] is a major postsynaptic scaffold protein that is thought to cluster postsynaptic NMDA receptors at postsynaptic sites. Most importantly, PSD-95, through interactions with transmembrane AMPA receptor regulatory proteins (such as stargazin) is believed to dictate the number of AMPA receptors found within the postsynaptic membrane (reviewed in [30]; see also [31]–[33]). Fluorescently tagged PSD-95 was used previously to study excitatory synapse formation (for example, [22],[34]–[39]), PSD turnover (for example, [40]–[42]), and PSD remodeling [22],[43], and was shown to faithfully represent PSD architectural rearrangements [43]. The particular EGFP-fusion protein used here was characterized extensively [35],[44]; When expressed in cultured hippocampal neurons, it was shown to localize correctly to postsynaptic structures associated with functional presynaptic sites, colocalize with the AMPA receptor subunit GluR1, and only negligibly affect gross synaptic characteristics [35]. In the current study, we used a third-generation lentiviral expression system [45] to express this fusion protein after an extensive series of preliminary experiments showed that this method was greatly preferable over more common transfection methods (calcium phosphate, cationic lipids, and electroporation): Lentivirus-based expression was easily titratable, resulted in low and constant expression levels, and importantly, unlike the aforementioned transfection methods, did not affect network activity properties or reduce cell numbers. Transduction was performed on day 5 in vitro leading to PSD-95:GFP expression in a small (10 to 50) number of neurons in each dish (and, occasionally, in nonneuronal cells). As shown in Figure 1, PSD-95:GFP assumed a punctate appearance, with the puncta commonly located at the tips of dendritic spines.

**Figure 2:**
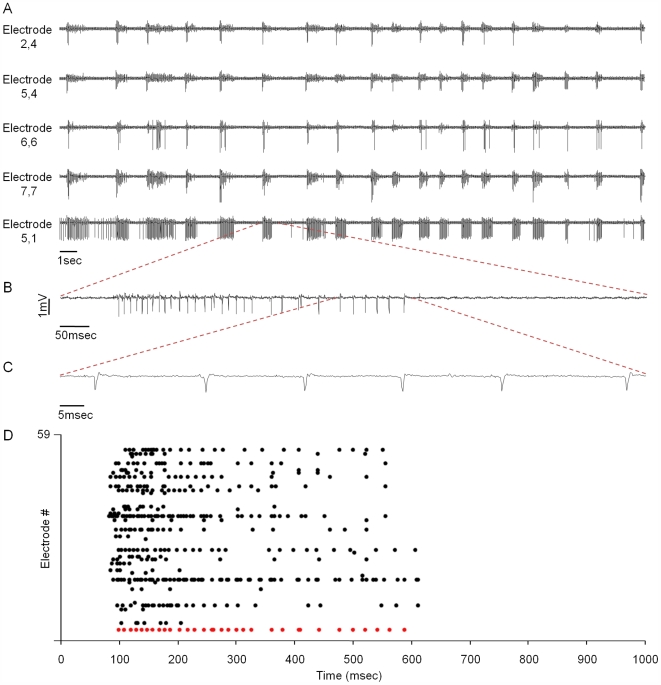
MEA-based recordings of spontaneous activity in cultures of cortical neurons. (A) Continuous extracellular recordings from five of the 59 electrodes in a MEA dish. Network activity exhibits some sporadic firing but mainly synchronous bursts. (B and C) A segment of a recording at higher resolution showing (B) trains of action potentials, and (C) individual action potentials. (D) Raster plots of action potentials recorded from 59 electrodes. Each action potential event is denoted as a single dot. The bottom row (red) corresponds to the raw data trace shown in (B).

To allow for long-term (many days) combined optical/electrophysiological recordings from these preparations, a commercial MEA headstage/amplifier was installed on a custom-built confocal laser scanning (inverted) microscope (CLSM) equipped with a robotic XYZ stage. In each experiment, the MEA dish was covered with a custom-built cap and placed in the headstage/amplifier connected to the CLSM's robotic stage. The MEA dish and oil-immersion objective were heated to 37°C, and a sterile mixture of 5% CO_2_, 95% air was streamed into the MEA dish. An ultraslow perfusion system was used to exchange the media at very low rates (two volumes per day). Images were collected automatically at 30-min intervals from four to 12 fields of view (or sites; ∼95×70 µm in size), with each site representing a portion of the dendritic arbor of a different neuron (Figure 1). Seven to 26 *Z*-sections were collected at each site, beginning at a predetermined offset above the upper glass surface. To correct for focal drift, the focal plane of the upper glass surface was located automatically before collecting each image stack [35]. Network activity in the imaged networks was recorded from all 59 (extracellular) embedded electrodes (Figure 2). For each electrode, waveforms of individual action potentials were stored digitally and then converted into series of discrete events (Figure 2D). Under these conditions, preparations were routinely maintained on the microscope stage, recorded from and imaged continuously for many days and even weeks (Figure 1C and 1D).

The synaptic identity of PSD-95:GFP puncta was verified by labeling active presynaptic compartments in live neurons with fluorescent antibodies against the lumenal domain of the synaptic vesicle protein synaptotagmin-1 ([46]; see Materials and Methods for further details). As shown in Figure S1, >80% of PSD-95:GFP puncta were juxtaposed against functional synaptic vesicle recycling sites, in good agreement with prior measurements performed in cultured hippocampal neurons using the styryl dye FM 4–64 (83%; [35]). The high degree of colocalization along with the fact that labeling was based entirely on spontaneous activity strongly indicates that most PSD-95:GFP puncta represent bona fide glutamatergic synapses that are activated by spontaneous network activity.

MEA dishes allowed us to sample network activity from 59 locations in the network, but due to the presence of multiple neurons near each electrode, the identity of neurons from which activity was recorded remained obscure. Furthermore, due to the random nature of lentiviral infection, neurons expressing PSD-95:GFP were not necessarily located over any particular electrode. It was thus necessary to verify that the activity recorded through the electrodes faithfully represented the activity of those neurons expressing PSD-95:GFP and followed by time-lapse microscopy. To that end, we took advantage of the fact that most activity in networks of dissociated cortical neurons occurs in the form of synchronized bursts (for example, [25],[27],[47]–[49]; see also Figure 2A–2C). By using fluorescent calcium indicators and synchronized MEA recordings, we found that practically all network bursts were time-locked to calcium transients measured by line scanning in the somata of PSD-95:GFP-expressing neurons (Figure S2; 27 neurons, four separate experiments). These experiments strongly indicate that the characteristics of network activity recorded through the MEA faithfully represent, at least to a first approximation, the activity of PSD-95:GFP-expressing neurons. Furthermore, the tight correlation between network bursts and calcium transients suggests that these neurons respond well to excitatory synapse activation, implying that PSD-95:GFP expression does not severely impair glutamatergic synapse functionality.

In summary, the system described here allowed us to follow structural dynamics of individual and functional glutamatergic synapses at relatively high temporal resolutions and over many days while concomitantly recording network activity in the same preparations.

### Spontaneous Changes in Network Activity, PSD Number, and PSD Size

We initially set out to the examine the long-term stability of PSD-95:GFP puncta under baseline conditions, that is, without experimentally manipulating network activity. To that end, we used preparations maintained in culture for at least 17 d. At this stage, networks of rat cortical neurons are considered to be relatively mature, and beyond the phase of extensive dendrite extension and synapse formation. The preparations were mounted on the CLSM and followed by combined time-lapse imaging and electrophysiological recordings as described above. At the end of each experiment, spike counts per electrode were extracted from the electrophysiological recordings, and the numbers of PSD-95:GFP puncta and their respective fluorescence intensities were extracted from maximal intensity projections of *Z*-stacks for each time point and each site (see Materials and Methods).

**Figure 3:**
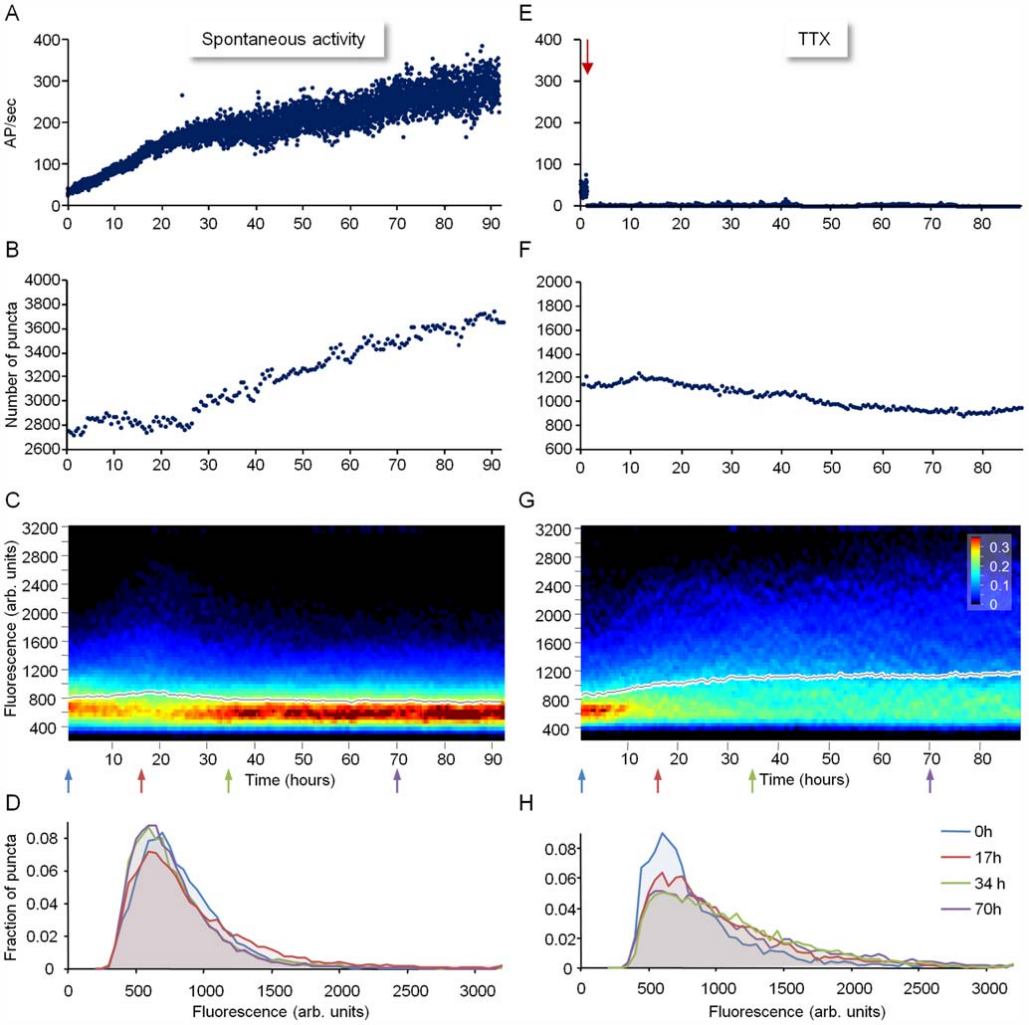
Evolution of network activity and PSD-95:GFP puncta population properties. (A) Spontaneous activity recorded for 93 h from a network of cortical neurons growing on a MEA dish, starting with the mounting of the preparation on the combined MEA recording/imaging system. Activity is expressed as action potentials (measured from all electrodes) per second. (B) Changes in the number of discernable PSD-95:GFP puncta in the same preparation during the same recording period (eight neurons). (C) Normalized distribution of fluorescence intensities of all discernable PSD-95:GFP puncta at each time point (bin size = 50 gray-scale units). Each vertical line represents the fractional distribution of fluorescence intensities for that time point, color coded according to scale bar in (G). Mean puncta fluorescence is shown as a black line. (D) Four representative histograms (bin size = 50 gray-scale units) from the time points marked and color coded by arrows in (C). Note that the initial broadening of the distribution of PSD-95:GFP puncta fluorescence intensities is followed by a constriction of this distribution. Also note that the increased bursting activity starting at approximately 25 h (manifested in the increased variability in spike rates from one minute to the other) is associated with an increase in PSD-95:GFP puncta numbers. (E) A similar experiment to that shown in (A–D), except that here, TTX was added (arrow) to the MEA dish (and to the perfusion medium) an hour after mounting the preparation. (F) Changes in the number of discernable PSD-95:GFP puncta (five neurons). (G) Normalized distribution of fluorescence intensities of all discernable PSD-95:GFP puncta at each time point. (H) Four representative histograms from the time points marked and color coded by arrows in (G). Unlike the experiment shown in (A–D), the broadening of the PSD-95:GFP puncta fluorescence intensity distribution was not reversed.

These experiments resulted in two highly consistent observations. The first concerns network activity. As noted above, cultured networks of dissociated cortical neurons typically develop complex patterns of spontaneous activity composed of asynchronous action potentials and synchronized bursts (Figure 2A–2C). These forms of activity were observed here as well, as soon as recording was initiated. However, over the first 1–2 d, we consistently observed significant elevations of spontaneous activity levels (Figure 3A). These elevations reflected both increases in the number of “active” electrodes as well as increases in the frequencies of action potentials measured from individual electrodes (Figure S3). Intermittently, periods of “superbursts” (i.e., bursts of bursts; [49]) were recorded (see below), giving rise to significant variability in action potential counts from one minute to the next. This gradual increase in network activity at the beginning of the experiment was observed in practically all experiments regardless of preparation age, indicating that it was due, somehow, to the environmental conditions introduced during the experiments (see below).

The second consistent observation concerns the morphological complexity of postsynaptic structures along dendritic segments. In practically all experiments, we observed a gradual increase in the total number of PSD-95:GFP puncta, mainly (but not exclusively) due to increased density of PSD-95:GFP puncta along existing dendritic segments, at both spine tips and shafts (Figures 1C and 3B). Moreover, the population of PSD-95:GFP puncta changed from one that was relatively uniform in terms of fluorescence intensity to one that contained both very large, bright puncta as well as many small, dim puncta (Figures 1C, 3C, and 3D). Interestingly, the broadening of the puncta intensity distribution was usually transient, and was partially reversed when network activity reached relatively high levels after 1–2 d (Figure 3A, 3C, and 3D). As the majority of PSD-95:GFP puncta were typically juxtaposed against functional presynaptic boutons (Figure S1; see also [35]), the observed increases in PSD-95:GFP puncta number reflected, in all likelihood, increased numbers of glutamatergic synapses. Furthermore, given that PSD size, PSD-95:GFP fluorescence, spine head dimensions, AMPA receptor number, and glutamate-induced synaptic currents, are well correlated [16],[50]–[54] changes in PSD-95:GFP content, probably reflected changes in PSD size and possibly in synaptic strength [8],[53].

The (post)synaptic remodeling described above resulted in dendrites assuming morphological characteristics more akin to those of dendrites in vivo. Yet we could not rule out the possibility that these morphological changes were actually reflecting pathological processes induced by the environmental conditions during experiments or damage inflicted by continuous imaging. To examine the possibility that the experimental conditions were detrimental to neuronal vitality, we used the same system to follow the development of less mature networks in which vigorous growth and synapse formation are known to occur, because here, pathological phenomena such as growth cessation, axon/dendrite retraction, and synapse elimination, are clearly recognizable. To that end, preparations were mounted on the CLSM starting from day 9–10 in vitro, maintained in the environmental conditions described above, and imaged at higher frequencies (every 10 min instead of 30) for about 1 wk. Dendritic development in these experiments appeared to proceed as expected: new branches were added, synapses were formed at high rates, and network activity levels increased 10- to 20-fold (Figure S4 and Video S1). In none of these experiments (*n* = 7) did we observe signs of damage. In fact, these experiments resulted in exciting and, to the best of our knowledge, unprecedented recordings of dendritic development and synapse formation that will be described elsewhere. These experiments, therefore, do not support the possibility that the experimental conditions used here adversely affect neuronal viability, and lead us to conclude that the synaptic remodeling described above is not secondary to pathological processes.

### Relationships between Network Activity and the Population Distribution of PSD Size

In the aforementioned experiments, we observed significant changes in PSD-95:GFP puncta number and fluorescence intensity, which, in all likelihood, reflected changes in glutamatergic synapse number and PSD size. This remodeling occurred concomitantly with significant changes in network activity, which pointed to the possibility that the two phenomena might be causally related. It should be noted, however, that unlike network activity, that generally increased over time (Figure 3A), the initial broadening of the PSD-95:GFP fluorescence intensity distribution (and the gradual increase in mean puncta fluorescence) was usually followed by a second phase during which the intensity distribution partially recovered (as did mean puncta fluorescence; Figure 3C and 3D) and thereafter remained stable for days, indicating that the potential relationships between the two phenomena are not straightforward.

To determine whether the observed changes in PSD-95:GFP puncta number and fluorescence intensity were dependent on changes in network activity, we repeated the experiments described above except that here, spontaneous network activity was blocked by adding tetrodotoxin (TTX) about an hour after the experiments were started. As shown in Figure 3E–3H, blocking network activity did not block the initial broadening of the PSD-95:GFP fluorescence intensity distribution. However, the second phase (the partial recovery of the intensity distribution) was completely lost. Instead, the distributions of PSD-95:GFP puncta fluorescence intensities continued to broaden, and mean puncta fluorescence continued to increase (Figure 3G and 3H). In addition, the number of puncta did not increase over time, and in fact, gradual decreases in puncta numbers were observed (Figure 3F).

These experiments indicate that the initial broadening of PSD-95:GFP puncta fluorescence intensity distribution is not driven by activity. Rather, it seems to be driven by the exposure to environmental conditions during experiments. Given that ambient temperature and atmospheric conditions were identical to those in the incubators in which preparations were maintained, the most likely “culprit” is the slow perfusion. Indeed, these phenomena are not observed if perfusion is not applied (unpublished data). On the other hand, in the absence of perfusion, the long-term viability of these preparations was drastically impaired. Interestingly, media turnover rates (∼0.15%/min) were one to two orders of magnitudes lower than cerebrospinal fluid (CSF) turnover rates in the intact rat brain (1% to 16%/min; [55]), indicating that perfusion rates were not excessively high.

In contrast to the initial broadening of PSD-95:GFP fluorescence intensity distributions, the subsequent constriction of fluorescence intensity distributions was clearly dependent on network activity. This dependence indicates that an increase in activity levels is associated with a reduction in mean PSD size. This finding is consistent with the concept of “synaptic scaling” [56]–[58], that is, the adjustment of synaptic strength to match neuronal activation levels. Interestingly, following the initial broadening and subsequent constriction, PSD-95:GFP puncta fluorescence intensity distributions remained relatively stable as long as activity levels did not change significantly (as exemplified in Figure 3C). These observations are consistent with the possibility that PSD size is generally stable, with changes in activity followed by uniform and gradual scaling of PSD size (multiplication by a scalar, for example). However, as shown next, this does not seem to be the case.

### Relationships between Network Activity and the Remodeling of Individual Postsynaptic Structures

The analysis described so far indicates that PSD size distribution remains rather stable as long as activity levels are not drastically altered. However, this analysis was performed at the population level, and thus did not provide information on the long-term stability of individual PSD sizes. To quantify the stability of individual PSDs, we developed software for tracking identified PSD-95:GFP puncta in long time series of image stacks, and used it to quantify the fluorescence of individual PSD-95:GFP puncta at a temporal resolution of 30 min over several days. Although PSD-95:GFP puncta were relatively stable over time scales of several hours, some puncta exhibited considerable dynamics (lateral movements, merging, and splitting) as previously described [22],[35],[36]. We therefore limited our analysis to PSD-95:GFP that could be identified and tracked reliably throughout the experiments, excluding puncta that became obscured by, merged with, or split from other puncta, but not excluding puncta that simply appeared or disappeared during the experiments. The fluorescence of all tracked puncta at all time points was then measured, and these data were compared to network activity during the same period.

**Figure 4:**
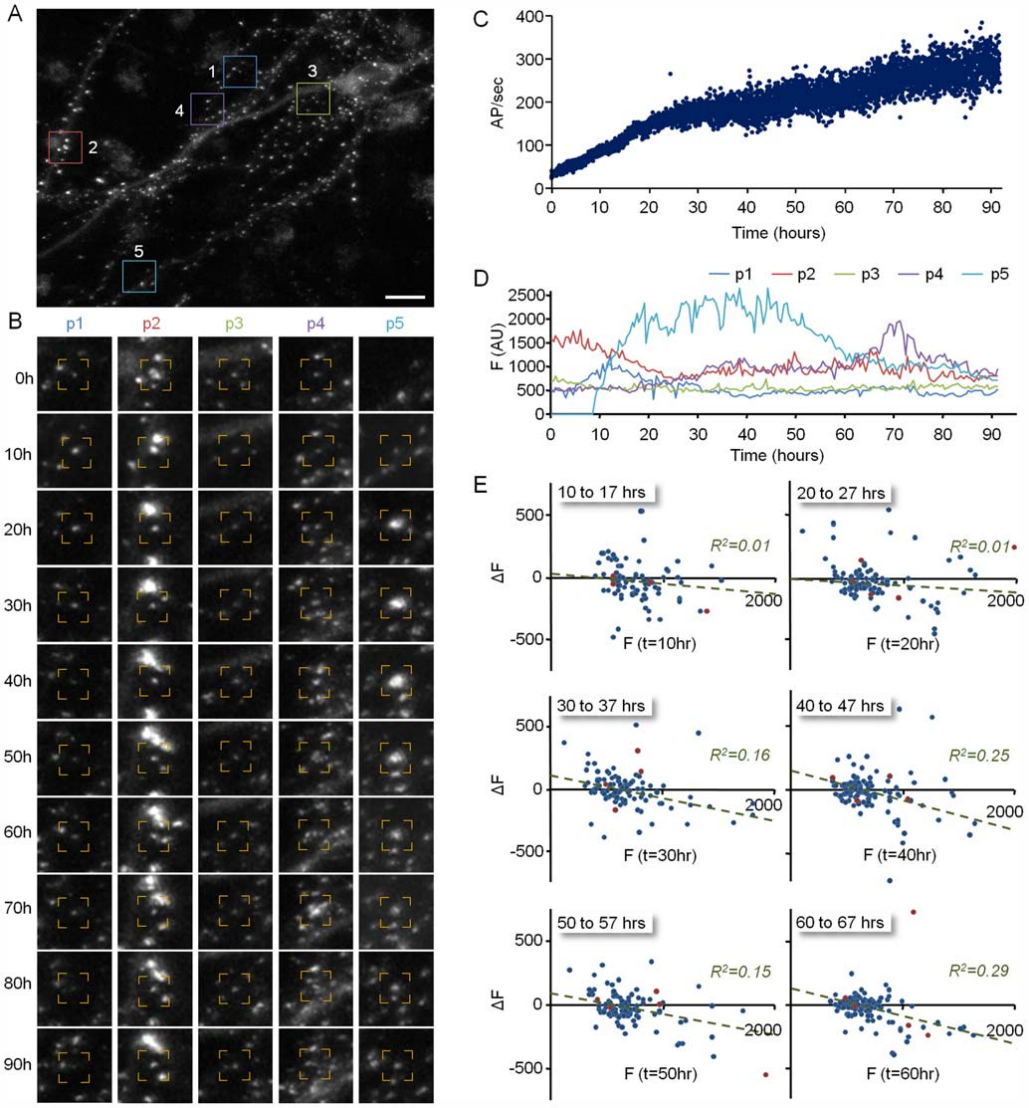
Remodeling of individual PSD-95:GFP puncta. (A) A neuron expressing PSD-95:GFP. (B). Five of the puncta (within color squares in [A]) that were tracked over the entire experiment. Note that it is not always apparent that the puncta along the time series are indeed the same ones. However, only one frame out of 20 sequential frames is shown, whereas imaging was performed at 30-min intervals, allowing for highly reliable tracking. (C) Evolution of network activity (same experiment shown in Figure 3A–3D) (D) Changes in the fluorescence of the five puncta shown in (A and B) over more than 90 h. (E) Changes in the fluorescence of individual PSD-95:GFP puncta as a function of their momentary fluorescence intensities at the beginning of 7-h time windows. Note that as activity levels increase, small synapses exhibit a tendency to grow larger, whereas large synapses exhibit a tendency to become smaller. Data from 201 synapses. Bar in (A) indicates 10 µm.

Figure 4 shows such an analysis performed for one neuron of the experiment of Figure 3A–3D. Five (out of ∼200) puncta tracked over the entire experiment are shown in Figure 4B. Note that it is not always apparent that the puncta shown in each time series are indeed the same ones. However, only one frame out of 20 sequential frames is shown in this figure (10-h intervals), whereas imaging was performed at 30-min intervals, allowing for very reliable tracking of discernable objects (see also Figure 1D). Plotting the fluorescence of these five puncta over >90 h (Figure 4D) revealed that some puncta exhibited significant changes in their fluorescence over this period, whereas the fluorescence of others was more stable. Yet, when the fluorescence of all tracked puncta is rendered for long stretches of time (days), the instability of individual PSD-95:GFP puncta becomes strikingly apparent (Video S2). These observations indicate that the seemingly static size distributions of Figure 3 are, in fact, population steady states, with individual synapses within this population undergoing continual and extensive remodeling.

To examine the dependence of changes in PSD size on initial PSD size, changes in the fluorescence of individually tracked puncta at the end of consecutive, 7-h time windows, were plotted as a function of their fluorescence at the beginning of each time window. To minimize the effects of short-term fluctuations, data were first “smoothed” with a five–time point (2-h) kernel. As shown in Figure 4E, significant changes in puncta fluorescence over time were observed for all puncta, regardless of their initial size. Interestingly, however, as activity levels increased, a relationship developed between initial puncta fluorescence and subsequent changes in fluorescence: Bright puncta tended to become dimmer, whereas very dim puncta tended to become brighter, as if activity was driving the convergence of PSD sizes to some optimal value. These relationships could be approximated reasonably well by linear regression fits to the data. In should be noted, however that the *R* ^2^ values of these linear fits were not very high, suggesting that the direction and magnitude of PSD size change were only partially determined by their instantaneous size.

**Figure 5:**
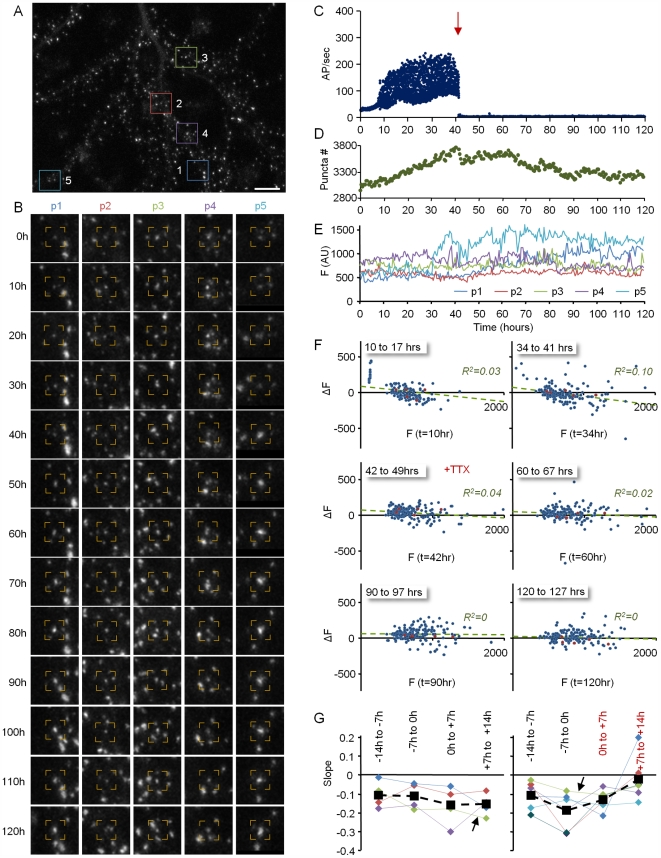
PSD-95:GFP puncta remodeling following abrupt termination of network activity. (A) A neuron expressing PSD-95:GFP. (B). Five of the puncta (within color squares in [A]) that were tracked over the entire experiment. (C) Evolution of network activity. Forty-two hours after the beginning of the experiment (red arrow), TTX was added to the MEA dish and the perfusion medium, resulting in the abrupt cessation of network activity. (D). Changes in the number of discernable PSD-95:GFP puncta in the same preparation (six neurons). (E) Changes in the fluorescence of the five puncta shown in (A and B) over 120 h. (F) Changes in the fluorescence of individual PSD-95:GFP puncta as a function of their momentary fluorescence intensities at the beginning of 7-h time windows. Note that after activity had ceased, changes in puncta fluorescence continued to occur. However, relationships between initial puncta fluorescence and subsequent changes in fluorescence were lost. Data from approximately 170 synapses (one neuron) tracked in one experiment. (G) Slopes of linear regression lines in four consecutive 7-h time windows before and after the addition of TTX (right; six neurons) or before an arbitrary time point (∼50 h; left; four neurons). Mean slopes are shown as black rectangles and dashed lines. Traces for experiments shown in Figures 4 and 5A–5F are marked with arrows. Bar in (A) indicates 10 µm.

To further examine the dependence of the aforementioned relationship on network activity, identical experiments were performed in which spontaneous network activity was blocked abruptly by adding TTX 40 to 70 h after the experiments were started. Significant changes in puncta fluorescence over time were still observed in the presence of TTX, and such changes were observed for small and large puncta alike (Figure 5 and Video S3). Strikingly, however, relationships between initial PSD-95:GFP puncta fluorescence and subsequent changes in fluorescence were lost. This is indicated by the fact that the slopes of linear regression lines fit to these data approached zero. Similar findings were observed for a total of six neurons (Figure 5G). As expected, TTX addition led to a broadening of the PSD-95:GFP puncta fluorescence distribution (Figure S5A). Furthermore, this was seen for both the entire PSD-95:GFP population and for the smaller population of tracked PSD-95:GFP puncta (Figure S5B), indicating that the population of tracked puncta faithfully represented the entire PSD-95:GFP puncta population.

**Figure 6:**
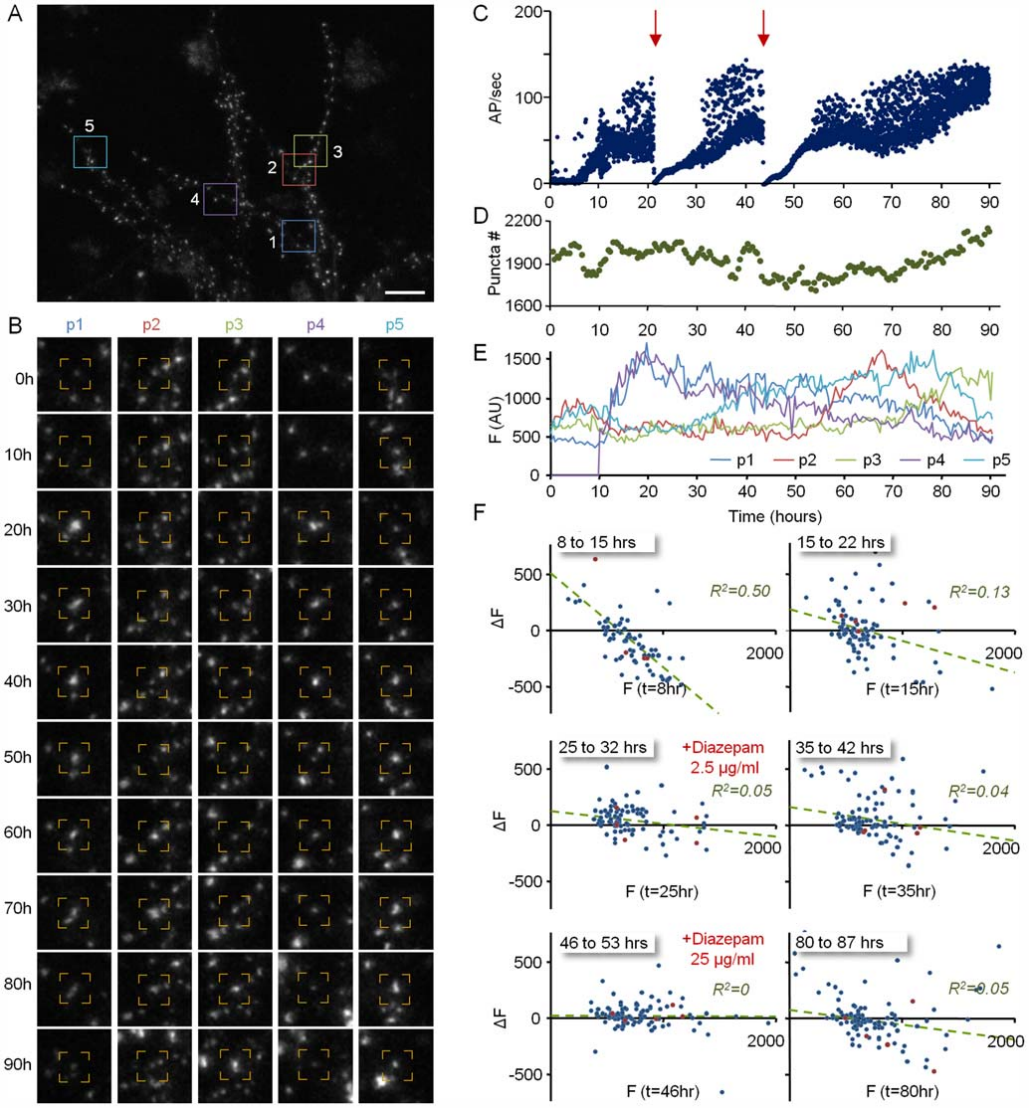
PSD-95:GFP puncta remodeling following abrupt suppression of network activity levels. (A) A neuron expressing PSD-95:GFP. (B) Five puncta (within color squares in [A]) tracked over the entire experiment. (C) Network activity in the same experiment. Twenty-two and 44 h after the beginning of the experiment (red arrows), diazepam (2.5 µg/ml and 25 µg/ml, respectively) was added to the MEA dish, resulting in temporary reductions in network activity levels. (D). Changes in the number of discernable PSD-95:GFP puncta in the same preparation (nine neurons). (E) Changes in the fluorescence of the five puncta shown in (A and B) over 90 h. (F) Changes in the fluorescence of individual PSD-95:GFP puncta as a function of their momentary fluorescence intensities at the beginning of 7-h time windows. Note that changes in puncta fluorescence occurred throughout the entire experiment. However, relationships between initial puncta fluorescence and subsequent changes in fluorescence were weakened or lost entirely following reductions in activity levels. Data from approximately 150 synapses (one neuron) tracked in one experiment. Bar in (A) indicates 10 µm.

Relationships between PSD-95:GFP puncta fluorescence and subsequent changes in puncta fluorescence were also examined by manipulating network activity with diazepam, a coagonist of GABA_A_ receptors in widespread clinical use. As shown in Figure 6, bolus additions of diazepam at two different concentrations (2.5 and 25 µg/ml) led to temporary and recoverable reductions in network activity levels (Figure 6C). Despite the reduction in activity levels, changes in puncta fluorescence still occurred to similar degrees, although the dependence of such changes on initial PSD-95:GFP puncta fluorescence was reduced or lost, in particular at higher diazepam concentrations (five experiments, tracked synapse data from four neurons).

**Figure 7:**
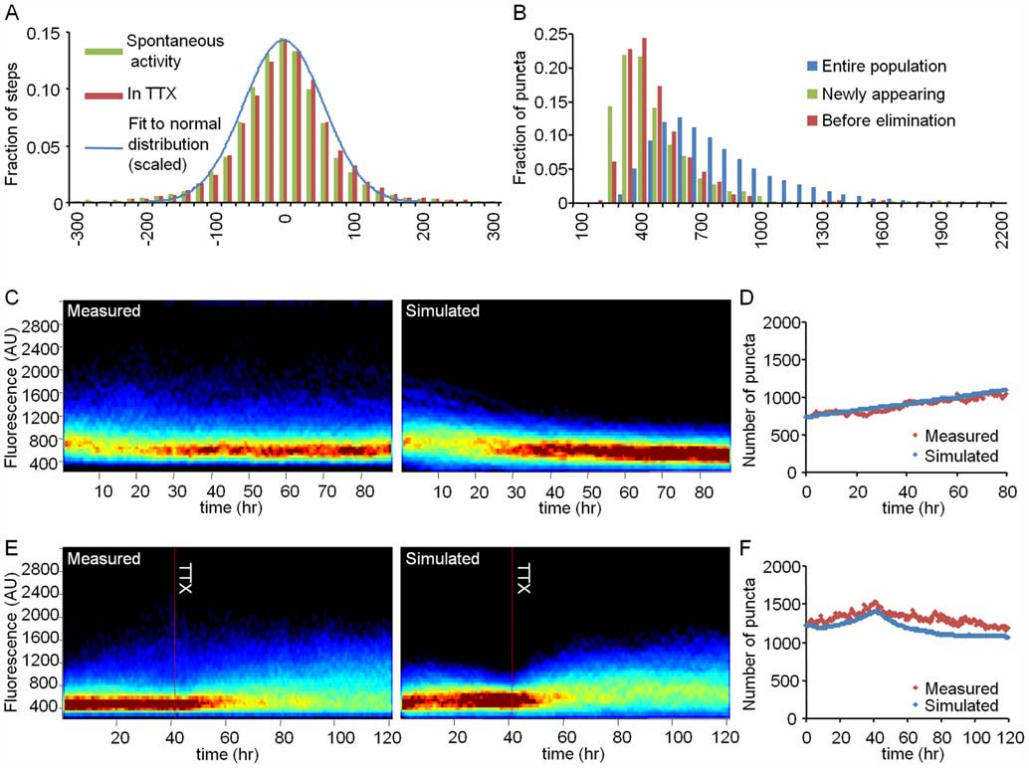
Modeling changes in the intensity distribution of PSD-95:GFP puncta. (A) Normalized histograms of single time step (30-min) changes in individual PSD-95:GFP puncta fluorescence collected for 20-h periods before and after the addition of TTX (17,716 and 20,056 steps, respectively; bin size = 20 fluorescence units). The data were fit to a (scaled) normal distribution curve (with a standard deviation of 56 fluorescence units). Note the similarity of the two distributions. (B) PSD-95:GFP puncta fluorescence distribution for puncta that had just appeared, just before they disappeared, and the entire puncta population (five experiments, 10 cells, 619 newly appearing synapses, 496 disappearing synapses, entire population size >10,000 synapses). Bin size = 75 fluorescence units. (C and E) Comparison of simulated and measured PSD-95:GFP puncta fluorescence distributions for the experiments shown in Figures 4 and 5. (D and F) Simulated and measured PSD-95:GFP puncta numbers in the same experiments. Simulations shown here were seeded using initial values of puncta counts and intensities measured of the experiments shown in Figures 4 and 5. At each time step, changes in puncta intensities were calculated as weighted sums of 1) a “drift”-related change: randomly assigned from the pool of fluorescence intensity differences measured in TTX (A), and 2) a momentary size-dependent change (see Materials and Methods). The weights used for calculating the weighted sum varied according to the normalized activity during this time step in the particular experiment being modeled (see Materials and Methods for further details). Thus, for example, if activity during this time step was very low, the weight of the current synaptic size in determining subsequent synaptic size would be very low as well, and the change would be mainly determined by the random “drift” value. If as a result of the calculated change, the punctum intensity fell below a predetermined threshold (set according to the dimmest detectable PSD-95:GFP puncta), it was eliminated. In addition, at each time step, new synapses were added at a constant rate with their initial intensities assigned randomly from the pool of newly appearing puncta intensity values (B). As evident in (B), newly appearing puncta were typically dimmer than “established” puncta (see also [17],[35]).

Several points are worth further emphasis. First, changes in puncta fluorescence continued to occur even when activity was suppressed or eliminated altogether. In fact, fluorescence intensity differences measured for individual PSD-95:GFP puncta between consecutive images distributed similarly for TTX-treated and untreated preparations (see Figure 7A). Second, even in active networks, instantaneous PSD size was not the sole determinant of subsequent changes in PSD size. This is evident, not only from the relatively low *R* ^2^ values of the linear regression fits, but also from the fact that the distribution of PSD sizes in active networks did not continue to constrict with time (Figure 3C and see also Figure S5A), indicating that activity-driven convergence of synaptic size distribution is balanced by processes that act to broaden PSD size distribution. Third, changes in the strength of relationships between instantaneous PSD size and subsequent changes in PSD size were slow to develop, and occasionally were only observed several hours after activity levels had changed significantly. Finally, although reductions in network activity levels were generally associated with increases in PSD size (Figure 5), we practically never observed (except one time point for one cell) a positive relationship between PSD size and the extent to which PSD size changed following reductions in activity levels. Put differently, once activity was blocked, changes in PSD size, in absolute terms, were similar for small and large PSDs alike (see Figure 5, 90–97 h, for example). This observation strongly argues against the possibility that increases in PSD size following reductions in activity levels can be viewed as simple multiplicative scaling.

### Modeling Relationships between Network Activity and Synaptic Remodeling

The long-term recordings of synaptic remodeling described so far indicate that (1) spontaneous network activity maintains distributions of synaptic sizes within rather constrained boundaries; (2) reductions in network activity levels result in a broadening of synaptic size distributions, increases in mean synaptic size, and reductions in synapse numbers; (3) most synapses exhibit significant changes in size over time; (4) in active networks, changes in synaptic size are partially dependent on momentary synapse size: large synapses tend to become smaller, whereas small synapses tend to become larger; and (5) when activity is blocked or significantly suppressed, synapses continue to change their sizes, but the direction and extent of these changes become independent of momentary synapse size.

We hypothesized that the phenomenological relationships between network activity and synaptic remodeling described above could be explained by the following set of rules: (1) synapses continuously undergo spontaneous, activity-independent changes (drift) in their size; (2) activity acts to reduce the size of large synapses on the one hand, and increase the size of small synapses on the other; (3) new, small synapses are continually formed at a constant rate; and 4) synapses whose size is reduced beneath some threshold are eliminated.

To examine whether this set of rules could, at least in principle, explain the phenomena described above and produce synaptic size distributions similar to those measured experimentally, we created a simple numerical model in which sizes and fates of individual synapses were updated over time according to these four rules (see legend of Figure 7 and Materials and Methods for further details). Figure 7C–7F shows simulations seeded with the initial puncta counts and intensities measured in the experiments of Figures 4 and 5. As these figures show, the numerical simulations recapitulated the major phenomenological relationships between network activity and synaptic size distributions (Figure 7C and 7E). Furthermore, they predicted correctly the changes in synaptic counts measured in these experiments (Figure 7D and 7F). These findings indicate that relationships between synaptic size distributions and network activity levels can be accounted for, at least in principle, by the four simple rules described above without a need for explicit “scaling” or compensatory mechanisms invoked by reductions in network activity levels (although this does not preclude their existence).

Although the simulated data generally approximated the experimental measurements quite well, they failed to account for a small population of particularly large synapses observed in active networks (visible as blue dots in the upper regions of the histograms of Figure 7C and 7E). We do not think this is related to imperfect simulation parameters but rather to the existence of at least one other “rule” that drives the formation of large synapses in highly active networks. Indeed, we noted that relatively large synapses tended to appear during periods of particularly high levels of synchronous activity, often associated with “superbursting” [48], observable as periods of significant variability in action potential counts from one minute to the other. In fact, a comparison of particularly bright (1.5 standard deviations above mean puncta fluorescence) PSD-95:GFP puncta appearance rates to burst rates revealed strong temporal relationships between these two phenomena (Figure S6A), relationships observed in practically all experiments in which superbursting occurred. In one striking example, tracking particularly bright puncta backward in time revealed that 18 out of 18 such bright puncta exhibited dramatic increases in fluorescence with the onset of superbursting or seemed to appear de novo (Figure S6B, S6C, and S6E; see also Figure 6, puncta 1 and 4). It is likely that these large PSD-95:GFP puncta represent synapses that underwent forms of potentiation associated with spine-head enlargement (e.g., [23],[53],[59]–[64]). In fact, we suspect that high levels of synchronized activity, rather than high activity levels per se are also the driving force behind the aforementioned tendency of small puncta to grow larger in highly active networks (see [65] and discussion below). As we did not have access to the precise firing timings of individual pre- and postsynaptic partners, this possibility remains somewhat speculative. Interestingly, however, as our system did allow us to follow these synapses for relatively long periods, we were able to compare their tenacity to that of the rest of the synaptic population. These observations indicated that newly enlarged synapses did not necessarily fair much better than other synapses in terms of their long-term tenacity, even in the presence of TTX (Figure S7; see also [23],[59]), although their lifespans were typically longer (unpublished data).

### Long-Term Stability of Synaptic Configurations

Individual CNS neurons, even those in our cell culture preparations, typically receive thousands of excitatory synaptic inputs of varying strengths. The experiments described so far reveal that individual PSD-95:GFP puncta exhibited significant remodeling over time, probably reflecting significant changes in synaptic strengths. Given the high levels of spontaneous activity in these networks, it might have been expected that this remodeling is driven to a large extent by network activity. However, as shown above, remodeling does not cease upon suppression or elimination of network activity. To what degree are the relative weights of excitatory inputs of a given neuron reconfigured by ongoing activity? How stable does this configuration remain when the physiologically relevant driving force, i.e., activity, is removed?

To evaluate the impact of synaptic remodeling on the synaptic configurations of individual neurons, we defined a measure for quantifying the degree to which PSD-95:GFP puncta belonging to a given neuron changed their sizes relative to each other over time (irrespective of global changes in puncta fluorescence). The basic idea was to sort the synapses formed on a given neuron according to their sizes. Then, at each subsequent time point, the same synapses were sorted again according to their new sizes. The degree to which each synapse changed its rank relative to its original rank was then determined, and finally, all rank changes for all synapses were summed and normalized to give a value between 0 and 1. This measure (denoted *M*_t_) was taken to represent the relative remodeling for that time point and for that particular neuron. In practice, *M*_t_ was calculated according to the following equation:
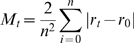
where *n* is the population of tracked PSD-95:GFP puncta, *r_t_* is its rank at time *t*, and *r_0_* its rank at time *t* = 0. *M_t_* will approach 1.0 if the rank of each synapse at time *t* is furthest away from its rank at time *t* = 0, and will approach 0.67 if the ranks at time *t* bear no relationships beyond chance to the ranks at *t* = 0.

**Figure 8:**
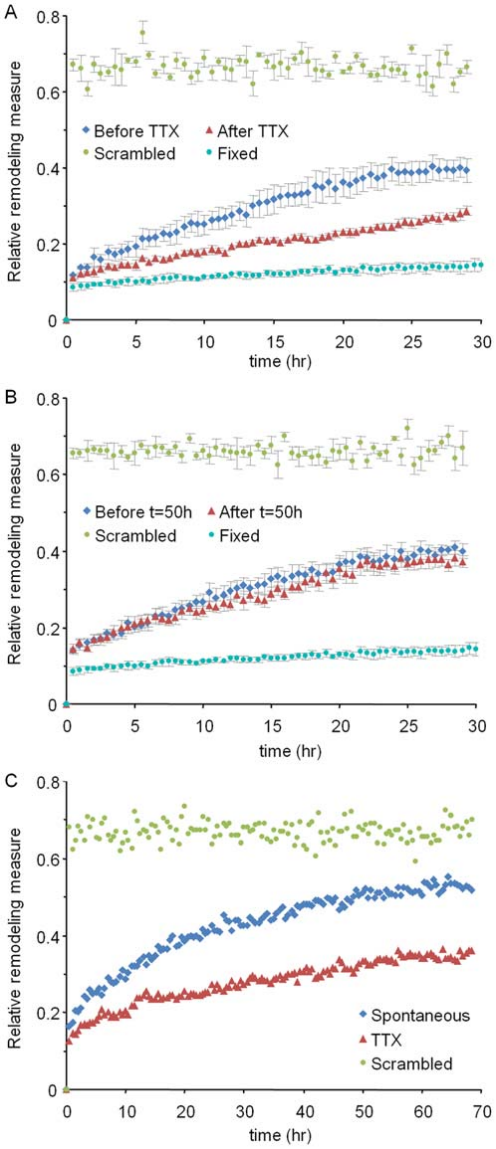
Relative remodeling rates in active and inactive networks. (A) Relative remodeling was determined for 30 h before and 30 h after the addition of TTX (four neurons). Note that TTX addition approximately halved the rate of synaptic remodeling, but remodeling did not cease. For comparison, measures obtained from scrambled (pre-TTX application) data are also shown, representing synaptic configurations that bear no relationship to the original configurations beyond those occurring by chance. The level of imaging-related artifacts can be estimated from identical measurements performed on paraformaldehyde-fixed neurons (three neurons). (B) Relative remodeling before and after an arbitrary time point (50 h; five neurons). (C) Relative remodeling measured for longer times (70 h) for a smaller number of neurons (three, and two, spontaneous activity and TTX, respectively). (A and B) show the means±standard error of the mean (SEM); (C) shows the means.

Figure 8A shows how the relative remodeling measure changes over 30 h before and 30 h after the addition of TTX (four different neurons, 505 PSD-95:GFP puncta). As shown in this figure, relative remodeling occurs about twice as fast in active networks as compared to networks in which activity was blocked. This effect is clearly activity dependent and not time dependent because no differences in relative remodeling rates are observed in two consecutive 30-h time windows when TTX is not added (Figure 8B). It is important to stress, however, that remodeling continues at significant, albeit slower rates even in the presence of TTX. This remodeling is not an artifact of imaging-related noise because practically no change in *M* was observed in control experiments performed in exactly the same experimental conditions using paraformaldehyde-fixed, PSD-95:GFP-expressing neurons (Figure 8A and 8B). Measurements made in a smaller number of neurons followed for longer periods (70 h, or about 3 d) further show that relative remodeling in this system is extensive, even in the presence of TTX, and that relative remodeling does not plateau within this time frame (Figure 8C).

These findings indicate that ongoing activity drives significant changes in the synaptic reconfigurations of individual neurons. Just as important, however, these findings also show that substantial “drift” in synaptic configurations occurs even in the absence of network activity, the physiologically relevant driving force.

## Discussion

Here, we describe experiments aimed at evaluating the long-term tenacity of individual glutamatergic synapses. To that end, we developed a novel system that allowed us to continuously follow and record the structural dynamics of synapses formed between rat cortical neurons in primary culture, over time scales of minutes to weeks, while concomitantly recording and manipulating network activity in the same preparations. We found that in spontaneously active networks, the range and distribution of synaptic sizes was maintained within rather constrained boundaries. Yet, when synapses within these populations were followed individually, the majority exhibited considerable changes in size over time scales of hours and days, and it became evident that the apparently static size distribution was in fact a steady state of synapses undergoing continual remodeling. Further analysis revealed that the extent and direction of this remodeling was partially dependent on momentary synaptic size, with large synapses exhibiting a tendency to grow smaller, and small synapses a tendency to grow larger. Blocking network activity did not stop synaptic remodeling, but changes in synaptic size became independent of momentary synaptic size. This undirected and unconstrained “drift” of synaptic size was associated with a broadening of synaptic size distributions, and gradual reductions in synaptic numbers. From the perspective of the single neuron, our experiments showed that activity drives changes in the relative weights of its excitatory inputs, but also revealed that these weights exhibit significant “drift” even in the absence of any network activity.

These findings point to several potentially important conclusions: First, they suggest that synaptic size exhibits continuous and significant “drift” over time (many hours to days) even in the absence of activity, indicating that the structural, and by extension, the functional tenacity of synapses is somewhat limited over long time scales. Second, they suggest that activity acts to partially direct this drift, promoting the convergence of synaptic sizes on some “optimal” size distribution. Third, although our findings do support previous reports that activity blockade is associated with increased synaptic size, they do not support the notion of multiplicative scaling [66]. Finally, our findings support the widespread belief that network activity drives synaptic remodeling and alters the relative weights of synapses formed on a given neuron. However, they also indicate that these relative weights undergo significant spontaneous, activity-independent changes as well.

### Concomitant Recording of Synaptic Remodeling and Network Activity

The experiments described here were based on several techniques: networks of dissociated cortical neurons, MEA substrates, automated multisite confocal microscopy, fusion proteins of synaptic proteins, lentiviral expression vectors, and automated image analysis. Although most of these techniques are in common use, it was their unique combination that allowed us to follow synaptic remodeling and relate it to network activity over relatively long time scales. Of particular note is the use of MEA substrates fabricated on very thin glass (ThinMEAs) that allowed the use of high numerical aperture objectives, resulting in both high-resolution images and very efficient light collection. In fact, control experiments performed in paraformaldehyde-fixed neurons revealed that photobleaching rates did not exceed 10% per day (48 time points per day, 15 focal planes per time point). This was undoubtedly an essential factor in our ability to image neurons at relatively high rates for such prolonged periods.

Another key technique was the use of an ultraslow perfusion system. This system maintained cell viability in a remarkable fashion: unlike typical long-term experiments carried out at physiological temperatures, where some rundown is usually observed after 12–24 h, we observed no signs of rundown even after 2 wk of continuous imaging. As the perfusion medium was identical to the normal growth medium, it would seem that medium replacement was the critical factor. Furthermore, we found that slow exchange rates were imperative, as rapid medium replacement was detrimental as well. A byproduct of the slow perfusion was a gradual increase in network activity resulting in very high and complex spontaneous spiking patterns. At present, we do not know why this occurs, but this phenomenon was instrumental in exposing the effects of activity and accentuating the effects of activity suppression.

A potential concern is the use of an exogenous form of PSD-95 fused to EGFP (an ∼30 kDa polypeptide). We cannot exclude the possibility that the addition of EGFP interferes, perhaps in subtle ways, with the interactions of PSD-95 with its endogenous binding partners, with implications on PSD remodeling dynamics. Furthermore, PSD-95:GFP overexpression was previously shown to affect synaptic properties and even occlude forms of activity-induced synaptic plasticity [67]–[71]. The severity of such effects, however, probably depends on overexpression levels. For example, in the first study mentioned above, PSD-95:GFP expression levels were reported to be many fold greater than endogenous PSD-95 levels, whereas the use of Sindbis or Semliki forest viral vectors for PSD-95:GFP expression in others might have resulted in similar situations [72]. In agreement with this possibility, other studies using fluorescently tagged variants of PSD-95:GFP did not detect significant effects on synaptic properties [54],[73],[74]. It should be noted that overexpression levels in our hands were low and similar to those we have previously reported (∼27%; [35]), and at these expression levels, effects on postsynaptic and presynaptic properties were very small [35]. Finally, in the current study PSD-95:GFP puncta did exhibit both “homeostatic” forms of synaptic remodeling as well as synchronous activity-driven remodeling, in agreement with studies based on immunohistochemistry, electron microscopy, relatively inert reporter molecules such as EGFP, or live imaging of AMPA receptors (for example, [53],[63],[73],[75]–[78]). Given the low PSD-95:GFP expression levels here and the fact that the aforementioned forms of synaptic plasticity were not occluded in our system, it seems unlikely that the phenomena described here are solely artifacts of PSD-95:GFP overexpression, although, as mentioned above, we cannot exclude the possibility of the introduction of some quantitative inaccuracies [79].

A broader concern relates to the fact that the study was performed in dissociated cell culture. Although it was this very fact that allowed us to concomitantly record synaptic remodeling and network activity as described above, we cannot ignore the possibility that the limited tenacity exhibited by synapses here is somehow related to this experimental system. We could mention the fact that many phenomena pertaining to synaptic dynamics described in cell culture were also observed in vivo (compare, for example, [80] with [54]). Nevertheless, it would be prudent not to take the absolute values provided here too literally. It is also important to note that the experiments were performed in neurons that are relatively immature (3–4 wk in vitro) as compared to those in the mature rat brain. Given that several measures of synaptic dynamics subside with age [15],[16],[54],[74], the absolute rates of synaptic remodeling reported here might overestimate those that occur in the mature brain. Yet it is worth noting in this regard that in vivo imaging indicates that spine-head remodeling is quantitatively similar in young and adult mice [15].

Finally, it is worth stressing that MEA recordings, unlike single-cell recordings used in other studies concerning activity-induced synaptic remodeling (for example, [53],[63]), do not allow one to directly relate changes in the structural properties of an imaged synapse to changes in the strength of the connection it mediates (see also [81]). However, the integrated system described here did allow us to study the remodeling of very large numbers (thousands) of individual postsynaptic densities over times scales of minutes to weeks and to explore how these dynamics are affected by measured levels of network activity, and hence expose long-term phenomena that become apparent only at the population level. Thus, in spite of the potential drawbacks raised above, the advantages offered by this system are substantial.

### Relationships between Network Activity and Synaptic Remodeling

Previous studies, initially in cell culture and later on in vivo as well, have shown that reductions in activity levels are followed by general increases in the strength of excitatory glutamatergic synapses, whereas enhanced activity levels have opposite effects (reviewed in [56]–[58]). It was suggested that these changes represent “homeostatic” mechanisms that serve to stabilize neuronal activity levels. Furthermore, it was shown that activity blockade-induced increases in synaptic strength were best explained by a scaling of synaptic strengths by the same multiplicative factor [66]. Finally, several studies [73],[78] have shown that synaptic scaling has a predominant, although not exclusive, postsynaptic component, manifested as an increase in the number of AMPA-type glutamate receptors localized to postsynaptic compartments.

Our findings that suppression of activity broadens the distribution of PSD-95:GFP puncta fluorescence intensities and increases mean PSD-95:GFP fluorescence (Figures 3 and 5) are in general agreement with the aforementioned studies (see also [77],[82]). Our findings, however, offer an alternative explanation for this phenomenon. First, these findings indicate that activity exerts a positive control on synaptic size, with changes in synaptic sizes inversely related to momentary synaptic size. Second, the findings indicate that the suppression of activity removes this positive control, resulting in unrestricted “drift.” These findings are consistent with the possibility that mean synaptic size thereafter increases because this drift is *asymmetrically constrained*: synapses grow both larger and smaller in apparently random fashion, but synapses that become too small are eliminated (see also [17]), further biasing the mean synaptic size toward larger values (Figure 7). Therefore, there might be no a priori need for mechanisms actively invoked by reductions in activity levels (although these might exist), that act to adjust synaptic size accordingly. Although the difference between negative signaling and the removal of positive signaling is somewhat semantic, it is a simpler explanation and thus, perhaps, more appealing.

An interesting finding was the observation that high levels of network activity were associated with gradual increases in PSD-95:GFP puncta numbers, whereas suppressed activity levels were associated with gradual decreases in PSD-95:GFP puncta numbers (see also [82],[83]). These phenomena could have resulted from changes in synapse formation rates, changes in synapse elimination rates, or both [40]. However, a third possibility exists: that high levels of activity, and in particular synchronous activity, promote the stabilization and growth of newly formed synapses, echoing the prominent role of synchronous activity in nervous system development [84]. Conversely, in the absence of such activity, the tendency of new synapses to be stabilized would be reduced, and thus more synapses would be lost. Indeed, recent work from De Roo and colleagues [23],[74] indicates that the vast majority of new synapses are transient, but activity, and in particular rhythmic activity, leads to the stabilization of new spines, possibly by promoting synaptogenic interactions with nearby axons [71] (see also [85]–[87]) and to their eventual enlargement. Given that most activity in our preparations was in the form of synchronized bursts, new synapses were probably more likely to be stabilized in active networks, as compared to networks in which activity was suppressed. By way of extension, synchronous activity may serve to promote the enlargement of a subset of small synapses—new and preexisting alike (Figure S6)—reducing the likelihood that their size will drift below some critical threshold resulting in their loss, and thus providing some explanation for the tendency we observed for small synapses to grow larger in active networks [65]. Furthermore, periods of highly synchronized activity often led to the appearance of large PSDs (Figure S6), in agreement with studies showing that synchronous activity and specific stimulation patterns lead to significant spine-head enlargement (for example, [23],[53],[59]–[64],[79]). Unfortunately, as mentioned above, MEA recordings did not allow us to relate changes in the structural properties of an imaged synapse to the specific activity patterns it experienced. Interestingly, however, we were able to follow the fate of such synapses, and we noted that in many cases, this enlargement was transient (Figure S7), in good agreement with similar observations recently made in cultured hippocampal slices [23]. We thus do not find strong evidence that the tenacity of such synapses is significantly different from that of other synapses.

High activity levels also promoted reductions in the size of large synapses (Figure 4). Given that synaptic size seems to be inherently limited [88], it is conceivable that larger synapses may be more sensitive to forces that limit synaptic size, and, by extension, by activity-driven, size-limiting forces. The exact nature of such activity-driven forces is unknown, but given the relatively long time scale of their actions, they are likely to involve protein exchange [54],[80],[89],[90], protein degradation [91], and local protein synthesis [92], although mechanisms activated on shorter time scales may also be involved [93]. Regardless of their specific nature, such processes would drive continuous change in synaptic sizes and lead to continuous reconfiguration of synaptic weights (Figure 8).

### Synaptic Drift and Synaptic Tenacity

The long-term recordings of individual PSD-95:GFP puncta described here indicate that synaptic sizes exhibit spontaneous changes over time scales of many hours and days. This is probably not an artifact of cell culture, as fluctuations in spine-head size from one day to the next were observed in vivo (for example, [14],[15],[17],[18]). Interestingly, in a study published very recently, Yasumatsu and colleagues [94] analyzed fluctuations in the volumes of individual dendritic spines in cultured rat hippocampal slices. In common with previous studies, a volume-filling dye (EGFP) and two-photon microscopy were used to image dendritic spines once a day for several days. However, this study also examined the effects of NMDA-type glutamate receptor antagonists on spine volume fluctuations and showed that such fluctuations are observed also in the presence of such antagonists. As network activity levels were not measured, actual activity levels remained unknown, and thus, relationships between spine size fluctuations and network activity remained speculative. Perhaps the fact that no synaptic homeostasis-like processes were detected may indicate that basal activity levels were rather low to start with. Nevertheless, these findings further support the possibility that synaptic remodeling might persist even in the absence of activity and that the findings reported here are not solely artifacts of dissociated cell culture.

Yasumatsu et al. [94] also proposed that distributions in spine size, rates of spine formation and elimination, and the long-term persistence of large spines might be explained by such fluctuations according to a mathematical model based on Brownian motion (or “random walk”) with drift and reflecting boundaries. Indeed, we also found that generally similar models can adequately describe synaptic size distributions in relatively quiescent networks (Figure 7). However, we found that such models fall short of providing satisfactory accounts of synaptic size distributions in highly active networks, and that these seem to be governed by additional principles, such as network synchronicity levels (Figure S6). Most importantly, the mere persistence of large spines (proposed to function as “write-protected” devices) would not guarantee synaptic configuration stability, in particular when considering that these exhibited the largest activity-independent volume fluctuations [94]. Thus, significant synaptic reconfiguration seems to be an unavoidable consequence of activity-independent synaptic remodeling.

When considering the extensive molecular dynamics occurring in the minute synapse, the limited long-term tenacity of individual PSDs might not seem surprising. Perhaps it may be unrealistic to expect that a biological structure with the dimensions of a PSD, composed of dozens to hundreds of copies of individual proteins, whose function depends on even smaller numbers of molecules and is located at extreme distances from the soma, will maintain its structural and functional properties with pinpoint precision for many months and years [12]. Consequently, and assuming that our findings are not unique to the experimental system used here, several tentative conclusions may be drawn.

The first conclusion concerns the significance of individual synapses in “encoding” some functional characteristic of a neuronal network. Obviously, the weight of individual excitatory synapses in determining postsynaptic neuron output is rather limited to start with (see, for example, [95]). However, our findings indicate that the reliability of these weights, already challenged by fluctuations on short time scales (release failures, quantal fluctuations), is further challenged by processes occurring over longer time scales. This finding indicates that if indeed persistent changes in network function are realized by changes in synaptic weights [1],[2], the weights of many synapses might need to be changed to produce an enduring alteration of network function.

The second conclusion concerns the possibility that the limited tenacity of synapses, the “drift” they exhibit, is actually a fundamentally important feature of synapses, in particular in developing networks. One could imagine that synaptic drift and the consequential drift of network function constitutes an “exploratory” process that allows networks to explore the space of synaptic configurations in search of appropriate synaptic input levels or functionally “useful” or “successful” configurations. Following this logic, configurations that produce desirable results would be stabilized, perhaps by diffuse neuromodulatory systems activated by salient stimuli or “rewards,” input that is obviously absent from our preparations. Although entirely speculative at this point, it is intriguing to consider the possibility that the principles of diversity and selection, arguably the most universal principles of evolving biological systems, might also be fundamental principles in the processes that govern synaptic remodeling.

## Materials and Methods

### Cell Culture

Primary cultures of rat cortical neurons were prepared in a similar manner to that described previously for hippocampal preparations [35]. Cortices of 1–2-d-old Sprague-Dawley rats were dissected, dissociated by trypsin treatment followed by trituration using a siliconized Pasteur pipette. A total of 1–1.5·×10^6^ cells were then plated on thin-glass MEA dishes (MultiChannelSystems MCS), whose surface had been pretreated with Polyethylenimine (Sigma) to facilitate cell adherence. Cells were initially grown in medium containing minimal essential medium (MEM; Sigma), 25 mg/l insulin (Sigma), 20 mM glucose (Sigma), 2 mM l-glutamine (Sigma), 5 µg/ml gentamycin sulfate (Sigma), and 10% NuSerum (Becton Dickinson Labware). The preparation was then transferred to a humidified tissue culture incubator and maintained at 37°C in a gas mixture of 5% CO_2_, 95% air. Half the volume of the culture medium was replaced three times a week with feeding medium similar to the medium described above but devoid of NuSerum, containing a lower l-glutamine concentration (0.5 mM) and 2% B-27 supplement (Invitrogen).

### FU(PSD-95:EGFP)W Plasmid Construction

FU(PSD-95:EGFP)W was assembled from FUGW [45], GW1-PSD95-EGFP-N3 (a generous gift by David Clapham; [44]), and pEGFP-N3 (Clontech Laboratories) in the following manner: PSD-95 was cut out of GW1-PSD95-EGFP-N3 using Nhe1 and HindIII. pEGFP-N3 was linearized using HindIII and SmaI. Linearized pEGFP-N3 and the PSD-95 fragment were ligated, and PSD-95:EGFP was subsequently cut out using XbaI and NheI. FUGW was linearized and the GFP fragment removed using XbaI. The process was completed by the ligation of the remaining FUGW and PSD-95:EGFP.

### Lentivirus Production and Transduction of Cortical Cultures

Lentiviral particles were produced using a mixture of FU(PSD-95:EGFP)W and the Lentiviral packaging vector mix of the ViraPower four-plasmid lentiviral expression system (Invitrogen). HEK293T cells were cotransfected with a mixture of FU(PSD-95:EGFP)W and the three packaging plasmids: pLP1, pLP2, and pLP\VSVG. Transfection was performed in 10-cm plates when the cells had reached 80% confluence, using 3 µg of FU(PSD-95:EGFP)W, 9 µg of the packaging mixture, and 36 µl of Lipofectamine 2000 (Invitrogen). Supernatant was collected after 48 and 72 h, filtered through 0.45-µm filters, aliquoted, and stored at −80°C. Transduction of cortical cultures was performed on day 5 in vitro by adding 5–15 µl of the filtered supernatant to each MEA dish.

### Electrophysiology

The thin-glass MEA dishes used here contained 59 30-µm-diameter electrodes arranged in an 8×8 array, spaced 200 µm apart. The dishes contain 59 electrodes, rather than 64, because the corner electrodes are missing, and one of the remaining leads is connected to a large substrate-embedded electrode designed for use as a reference (ground) electrode. The flat, round (30-µm diameter) electrodes are made of titanium nitride, whereas the tracks and contact pads were made of transparent indium tin oxide (Figure 1A).

Network activity was recorded through a commercial 60-channel headstage/amplifier (Inverted MEA1060; MCS) with a gain of X1024 and frequency limits of 1–5,000 Hz. The amplified signal was multiplexed into 16 channels, amplified by a factor of 10 by a 16-channel amplifier (Alligator Technologies), and then digitized by an A/D board (Microstar Laboratories) at 12 K samples/s per channel. Data acquisition was performed using AlphaMap (Alpha-Omega). Most data were stored as threshold-crossing events with the threshold set to −30 µV. Electrophysiological data were imported to Matlab (MathWorks) and analyzed using custom-written programs.

### Long-Term Imaging

Scanning fluorescence and brightfield images were acquired using a custom-designed confocal laser scanning microscope based on a Zeiss Aviovert 100 using a 40× 1.3 NA Fluar objective. The system was controlled by software written by one of us (NEZ) and includes provisions for automated, multisite time-lapse microscopy [35]. MEA dishes containing networks of cortical neurons were mounted in the headstage/amplifier that was attached to the microscope's motorized stage. The MEA dish was covered with a custom-designed cap containing inlet and outlet ports for perfusion and air, a reference ground electrode (a circular platinum wire), and a removable transparent glass window. The MEA dish was continuously perfused with feeding medium (described above) at a rate of 5 ml/d by means of a custom-built perfusion system based on an ultra-low-flow peristaltic pump (Instech Laboratories) using an imbalanced set of silicone tubes. The tubes were connected to the dish through the appropriate ports in the custom-designed cap. A mixture of 95% air with 5% CO_2_ was continuously streamed into the dish at very low rates through a third port, with flow rates regulated by a high-precision flow meter (Gilmont Instruments). The base of the headstage/amplifier and the objective were heated to 38°C and 36°C, respectively, using resistive elements, separate temperature sensors, and controllers, resulting in temperatures of approximately 37°C in the culture medium.

EGFP was excited by using the 488-nm line of an argon laser. Fluorescence emissions were read through a 500–545-nm bandpass filter (Chroma Technology). Time-lapse recordings were usually performed by averaging six frames collected at each of seven to 26 focal planes spaced 0.8–1 µm apart. All data were collected at a resolution of 640×480 pixels, at 12 bits/pixel, with the confocal aperture fully open. To increase experimental throughput, we collected data sequentially from up to 11 predefined sites, using the CLSM robotic XYZ stage to cycle automatically through these sites at 30-min time intervals. Focal drift during the experiment was corrected automatically by using the CLSM autofocus feature [35].

### Fura Red Calcium Imaging

Fura Red labeling of virally transduced cells was performed at the end of several long-term experiments. A total of 1 µl of Fura Red (Invitrogen) from a 2 mM stock solution in DMSO was diluted into 800 µl of culture medium drawn from the preparation dish, subsequently returning the mixture into the dish and mixing gently. After 30 min of incubation, line scan imaging through the neurons somata was performed at a rate of 54 lines/s. At the beginning of each scan cycle, a TTL signal was generated by the microscope and recorded to one of the four free channels of the electrophysiology data acquisition system for temporally aligning imaging and electrophysiological recordings performed during the same period. Fura Red was excited at 488 nm, and the emission was read through a 565-nm long-pass filter (Chroma Technology).

### Functional Labeling of Synaptic Boutons Using Anti-Synaptotagmin-1 Antibodies

The synaptic identity of PSD-95:GFP puncta was verified by labeling active presynaptic compartments with fluorescent antibodies against the lumenal domain of the synaptic vesicle protein synaptotagmin-1 [46]. These antibodies are taken up by synaptic vesicles whenever they undergo cycles of exocytosis and endocytosis, leading to their accumulation at active synaptic vesicle recycling sites. Labeling was performed as follows: monoclonal anti-synaptotagmin-1 antibodies (Synaptic Systems) were labeled with Alexa-647-tagged Fab fragments using the Zenon antibody labeling kit (Invitrogen). One microgram of the primary antibody was diluted in 18 µl of sterile PBS followed by the addition of 10 µl of IgG labeling reagent. The mixture was incubated in the dark for 5 min, after which, 10 µl of blocking solution was added followed by a second, 5-min incubation. The anti-synaptotagmin-1–Fab mixture was diluted in 500 µl of medium drawn from the MEA dish, and subsequently, the entire volume was returned to the dish and gently mixed. Time-lapse imaging was then continued for 6–12 h. Fluorescent puncta were observed approximately 20–30 min after the mixture application using the 633-nm line of helium-neon laser for excitation and a 640-nm long-pass emission filter (Chroma Technology).

### Pharmacological Manipulations

TTX (Alomone Labs) or diazepam (Teva) were diluted in 100 µl of medium drawn from the culture dish. The mixture was subsequently returned to the dish and mixed gently. The final concentration in the dish was 1 µM (TTX) or 2.5/25 µg/ml (diazepam). The addition of TTX to the dish was also followed by the addition of TTX to the perfusion medium.

### Imaging Data Analysis

All imaging data analysis was performed using custom-written software (“OpenView”) written by one of us (NEZ). For the purpose of this project, major portions of the software were rewritten to allow for automated/manual tracking of objects in 3-D time series of confocal images. Boxes of 8×8 pixels were centered on fluorescent puncta, and mean pixel intensities within these boxes were obtained from maximal intensity projections of *Z*-section stacks. For measuring distributions of puncta intensities (such as those of Figure 3), boxes were placed programmatically at each time step using identical parameters, but no tracking of individual puncta was performed. For tracking identified puncta, all puncta were initially boxed and then a smaller number of puncta (typically 200) were selected randomly and thereafter tracked. Automatic tracking was based on weighted comparisons of vicinity (in *X*, *Y*, and *Z*), intensity, and most importantly, “constellations,” that is, punctum location relative to neighboring puncta within a radius of 50 pixels. The reliability of automatic tracking was reasonable, but not perfect, and therefore, all tracking was verified and, if necessary, corrected manually.

All data were exported to Matlab and analyzed using custom-written algorithms. Images for figures were processed by linear contrast enhancement and low-pass filtering using Adobe Photoshop and prepared for presentation using Microsoft PowerPoint.

### Modeling

Changes in the intensity of a given PSD-95:GFP punctum were modeled as follows: at each time point *t*, the stepwise change in fluorescence Δ*f(t)* was calculated as a weighted (*w*, 1 − *w*) sum of a predictable, activity dependent component Δ*f_p_(t)* and a random component Δ*f_r_(t)*:
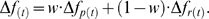



The relative weights of the two components were set to depend on the normalized spike rate *S(t)* (0 to 1):
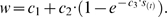



The predictable component was calculated as follows:
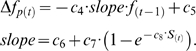



The random component was determined by randomly choosing a step from a pool of >20,000 measured steps (in TTX, starting 5 h after application; Figure 7A). New synapses were added at fixed rates. The initial size of new synapses was determined by randomly choosing a synapse from a pool of >600 experimentally measured new synapses (Figure 7B). A threshold was set based on the dimmest PSD-95:GFP puncta that could be detected in our system, and synapses whose fluorescence levels fell below this threshold were eliminated. Simulations were seeded using initial values of puncta counts and intensities measured in real experiments. All constants were maintained between all simulations, except *c1*, *c2*, and *c3*.

## Supporting Information

Figure S1 **PSD-95:GFP puncta represent functional synapses.** (A) A dendrite of a neuron expressing PSD-95:GFP. (B) Labeling of functional synapses with Alexafluor 647–tagged antibodies against the lumenal domain of synaptotagmin-1. The antibodies were added to the MEA dish, and spontaneous activity in the network led to the labeling of functional presynaptic boutons over several hours. (C) Overlay of images in (A and B). Note the good overlap of PSD-95:GFP and synaptotagmin-1–labeled presynaptic boutons. (D) Degree of match between PSD-95:GFP puncta and synaptotagmin-1–labeled presynaptic boutons as a function of time from antibody addition. Time point of image in (B) is shown in red.
(1.04 MB PDF)

Figure S2 **Imaging of Ca^2+^ transients in the soma of a cell expressing PSD-95:GFP.** (A) An X-t (line scan) image of Fura Red fluorescence at the cell body of a neuron expressing PSD-95:GFP. (B) Averages of fluorescence intensities in each line. Note that Ca^2+^ elevations *reduce* Fura Red fluorescence. (C) Raster plots of action potentials measured from all MEA electrodes over the same period. Each dot denotes a single action potential. (D) Total action potentials recorded from all electrodes in 1-ms bins. (E) Number of active electrodes over the same period. Note the tight time-locking between action potential bursts measured via the MEA and the calcium transients measured at the soma.
(0.18 MB PDF)

Figure S3 **Evolution of activity recorded from individual MEA electrodes.** Activity recorded from each electrode over the duration of an entire experiment (same experiment shown in Figures 3A–3D and 4). Activity is displayed as action potentials per second according to color scale at bottom.
(0.04 MB PDF)

Figure S4 **Long-term recordings of dendritic development.** (A) A dendritic segment of a cortical neuron expressing PSD-95:GFP was imaged continuously at 10-min intervals (seven sections per time point, 144 images/day) from day 10 to day 17 in vitro, (>6 d; only a small subset of the data is shown here). Time interval between the images shown here is 24 h. (B) Changes in PSD-95:GFP puncta numbers over time for three cells in this preparation (the cell shown in [A] is Cell 2). (C) Development of spontaneous activity in the same network. Note the concomitant increase in synaptic density and spontaneous activity levels. No obvious signs of phototoxicity or otherwise detrimental processes were observed. See also Video S1. Bar indicates 20 µm.
(1.21 MB PDF)

Figure S5 **Comparison of fluorescence intensity distributions for all PSD-95:GFP puncta and tracked puncta.** (A) Normalized distribution of fluorescence intensities of all discernable PSD-95:GFP puncta at each time point (same data as Figure 7E). (B) Normalized distribution of fluorescence intensities of all 281 tracked puncta in this experiment.
(0.03 MB PDF)

Figure S6 **Synchronous activity drives the appearance of particularly large synapses.** (A) Temporal correlations between burst rates and the appearance rates of bright synapses. Bright puncta were examined in a sliding time window of 5 h. A global threshold was defined (1.5 standard deviations above mean PSD-95:GFP puncta fluorescence). Puncta were counted if their brightness was at least 200 fluorescence units below the threshold at the beginning of the time window and exceeded the threshold at the end of the time window. Burst counts were smoothed with a 2-h kernel. Same experiment as that of Figure 4. (B) Eighteen bright PSD-95:GFP puncta at *t* = 22 h were tracked backwards in time to the beginning of the experiment. Each line denotes the fluorescence intensity of one punctum. Horizontal dashed line indicates mean+1.5 standard deviations. Same experiment shown in Figure 6. (C) Network activity levels during the same period. (D) Bursts rates for three 1-h periods in this experiment (red bars in [C]). Data were smoothed using a 60-s kernel.
(0.12 MB PDF)

Figure S7 **Relationships between changes in fluorescence in consecutive time windows, before and after addition of TTX.** Absolute changes in fluorescence (two top rows) and fractional changes (two bottom rows) in consecutive 10-h time windows straddling the moment of TTX application. Data are shown in raw form (first and third rows) and in normalized form: after correcting for the mean change of the entire population in each time window (second and fourth rows). This analysis indicates that most PSDs that had recently experienced considerable growth or shrinkage do not seem to be particularly protected from subsequent change upon complete activity blockade.
(1.05 MB PDF)

Video S1 **A week-long time-lapse recording of a developing dendrite.** Images were obtained every 10 min, seven sections per time point. Note the extraordinary developmental dynamics displayed by dendrites and axons in the field of view and the lack of any apparent signs of phototoxicity.
(10.54 MB MPG)

Video S2 **Changes in the fluorescence of individual PSD-95:GFP puncta over time.** Each circle represents one PSD-95:GFP punctum, with its momentary fluorescence depicted by its position along the *y-*axis. Data from two neurons, 349 synapses, and 114 30-min time steps. Data were smoothed with a six–time step kernel.
(2.48 MB MPG)

Video S3 **Changes in the fluorescence of individual PSD-95:GFP puncta over time in the presence of TTX.** Each circle represents one PSD-95:GFP punctum, with its momentary fluorescence depicted by its position along the *y-*axis. Data from two neurons, 281 synapses, and 110 30-min time steps. Data were smoothed with a six–time step kernel.
(2.48 MB MPG)

## Abbreviations

CLSM: confocal laser scanning microscope
MEA: multielectrode array
PSD: postsynaptic density
