# Supplementary figures and images for: Long-Term Relationships between Synaptic Tenacity, Synaptic Remodeling, and Network Activity
Source: PLoS Biol. 2009 Jun 23;7(6):e1000136. doi: 10.1371/journal.pbio.1000136 (PMC2693930; doi:10.1371/journal.pbio.1000136)

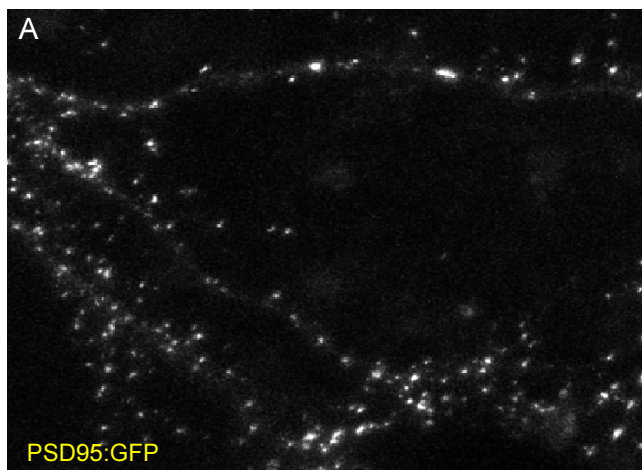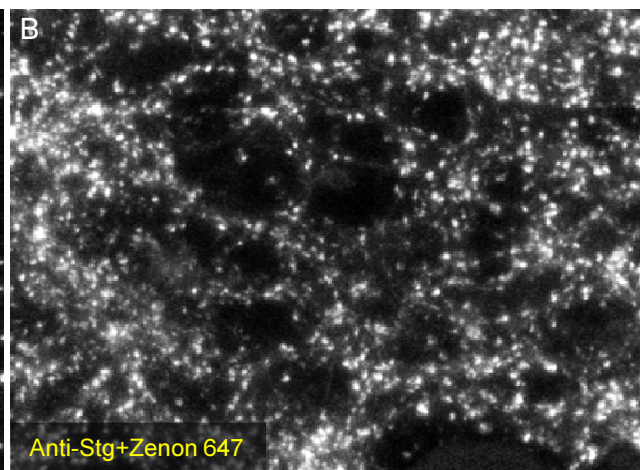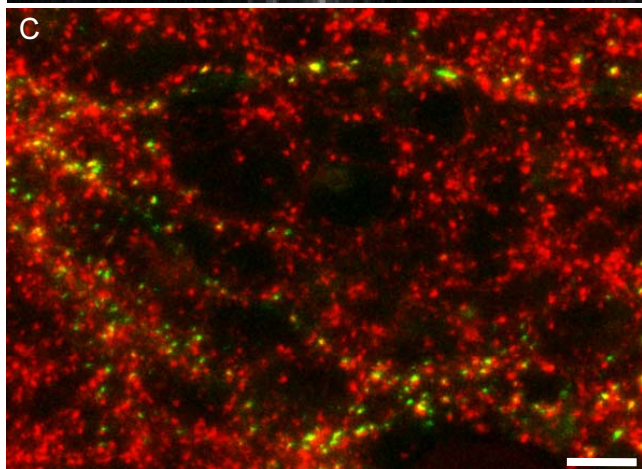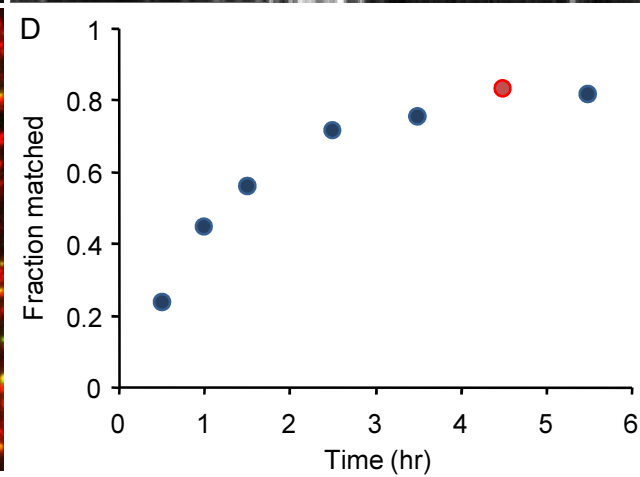

Supplement: Figure S1 — PSD-95:GFP puncta represent functional synapses. (A) A dendrite of a neuron expressing PSD-95:GFP. (B) Labeling of functional synapses with Alexafluor 647–tagged antibodies against the lumenal domain of synaptotagmin-1. The antibodies were added to the MEA dish, and spontaneous activity in the network led to the labeling of functional presynaptic boutons over several hours. (C) Overlay of images in (A and B). Note the good overlap of PSD-95:GFP and synaptotagmin-1–labeled presynaptic boutons. (D) Degree of match between PSD-95:GFP puncta and synaptotagmin-1–labeled presynaptic boutons as a function of time from antibody addition. Time point of image in (B) is shown in red. (1.04 MB PDF) [file pbio.1000136.s001.pdf]

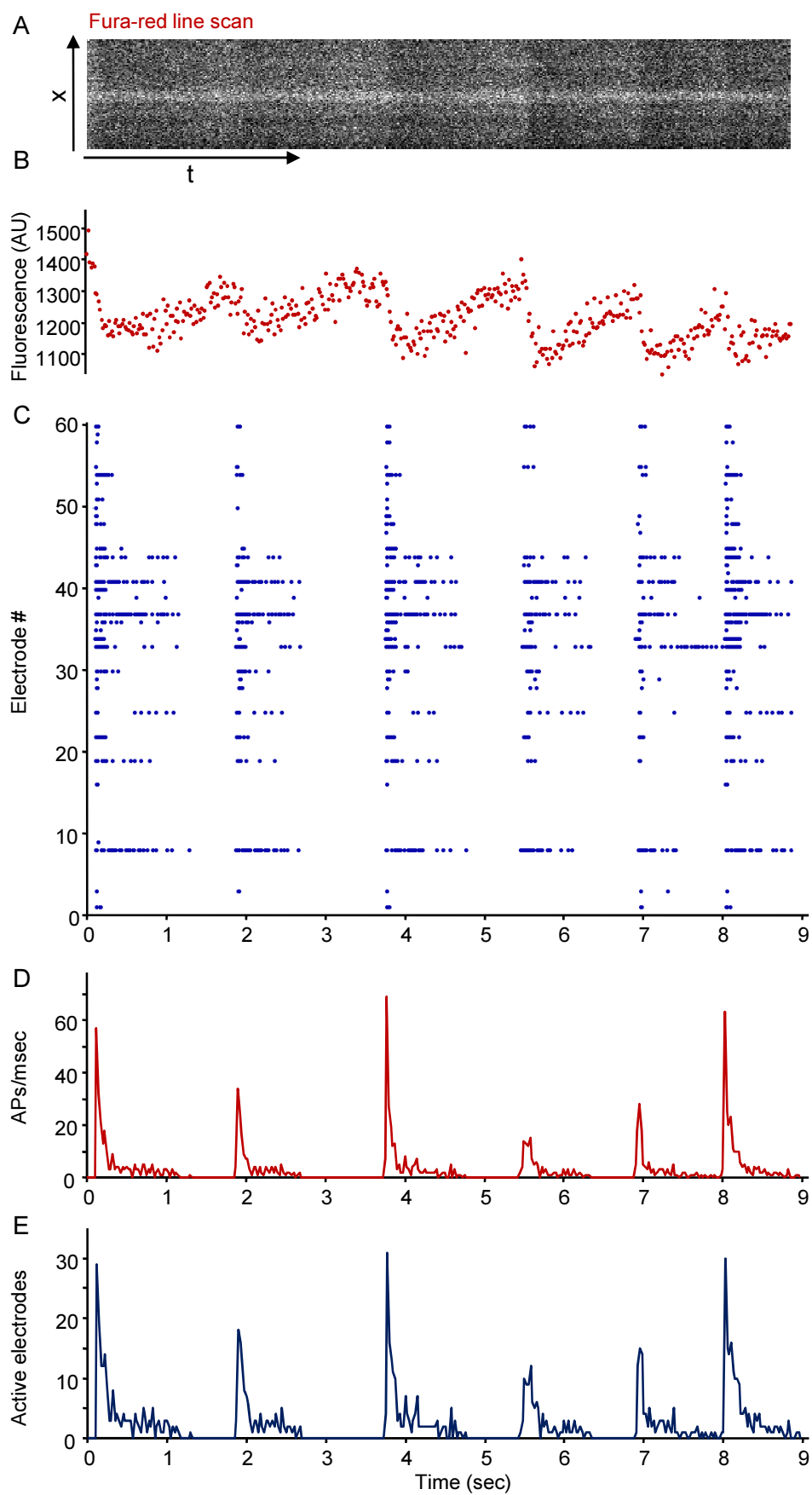

Supplement: Figure S2 — Imaging of Ca2+ transients in the soma of a cell expressing PSD-95:GFP. (A) An X-t (line scan) image of Fura Red fluorescence at the cell body of a neuron expressing PSD-95:GFP. (B) Averages of fluorescence intensities in each line. Note that Ca2+ elevations reduce Fura Red fluorescence. (C) Raster plots of action potentials measured from all MEA electrodes over the same period. Each dot denotes a single action potential. (D) Total action potentials recorded from all electrodes in 1-ms bins. (E) Number of active electrodes over the same period. Note the tight time-locking between action potential bursts measured via the MEA and the calcium transients measured at the soma. (0.18 MB PDF) [file pbio.1000136.s002.pdf]

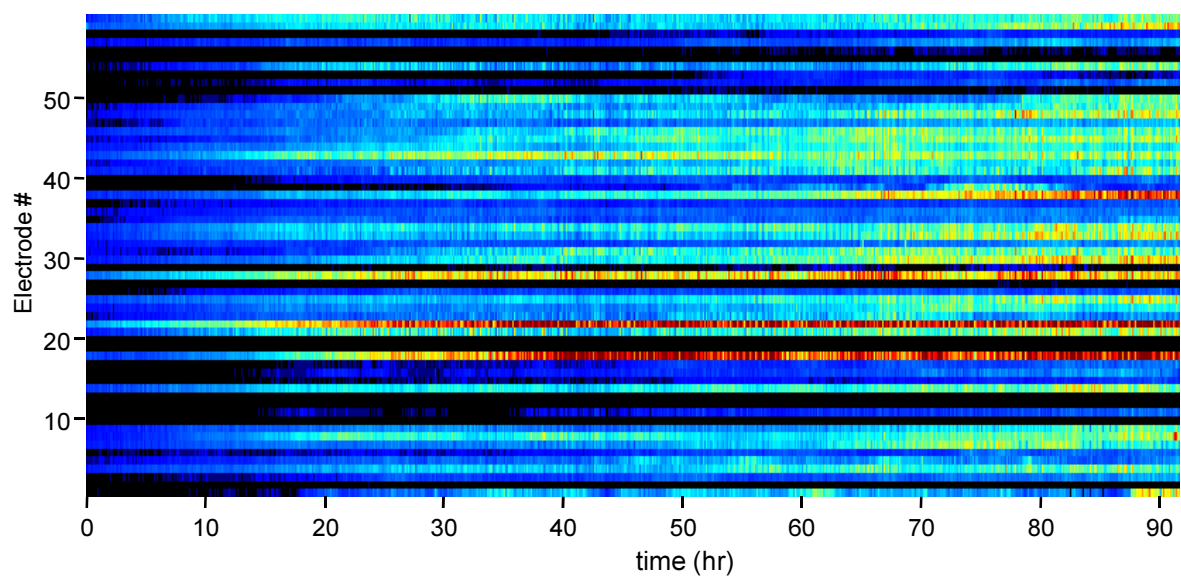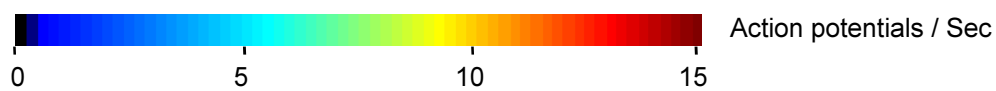

Supplement: Figure S3 — Evolution of activity recorded from individual MEA electrodes. Activity recorded from each electrode over the duration of an entire experiment (same experiment shown in Figures 3A–3D and 4). Activity is displayed as action potentials per second according to color scale at bottom. (0.04 MB PDF) [file pbio.1000136.s003.pdf]

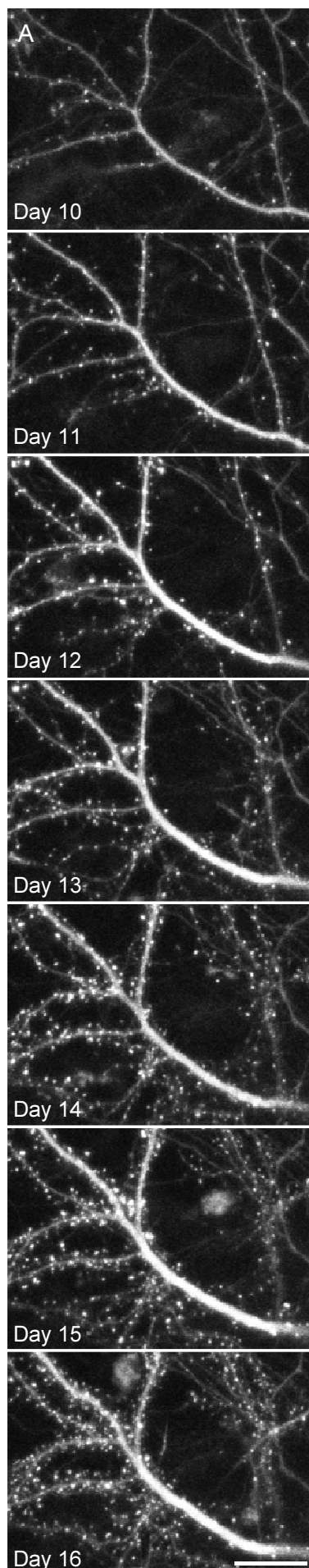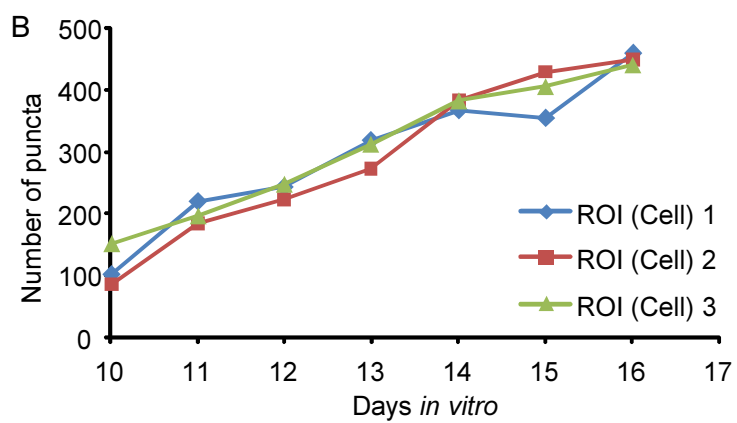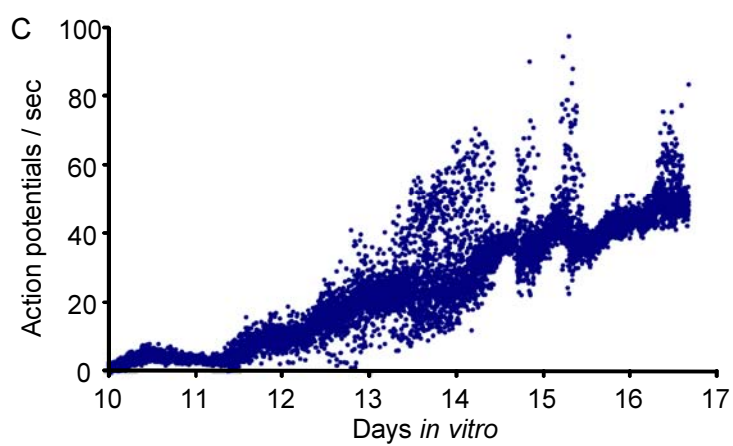

Supplement: Figure S4 — Long-term recordings of dendritic development. (A) A dendritic segment of a cortical neuron expressing PSD-95:GFP was imaged continuously at 10-min intervals (seven sections per time point, 144 images/day) from day 10 to day 17 in vitro, (>6 d; only a small subset of the data is shown here). Time interval between the images shown here is 24 h. (B) Changes in PSD-95:GFP puncta numbers over time for three cells in this preparation (the cell shown in [A] is Cell 2). (C) Development of spontaneous activity in the same network. Note the concomitant increase in synaptic density and spontaneous activity levels. No obvious signs of phototoxicity or otherwise detrimental processes were observed. See also Video S1. Bar indicates 20 µm. (1.21 MB PDF) [file pbio.1000136.s004.pdf]

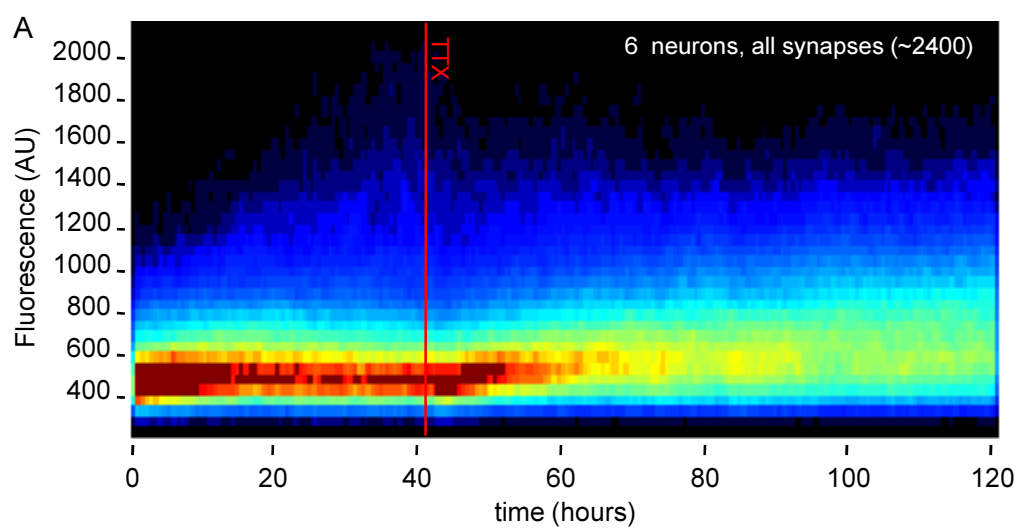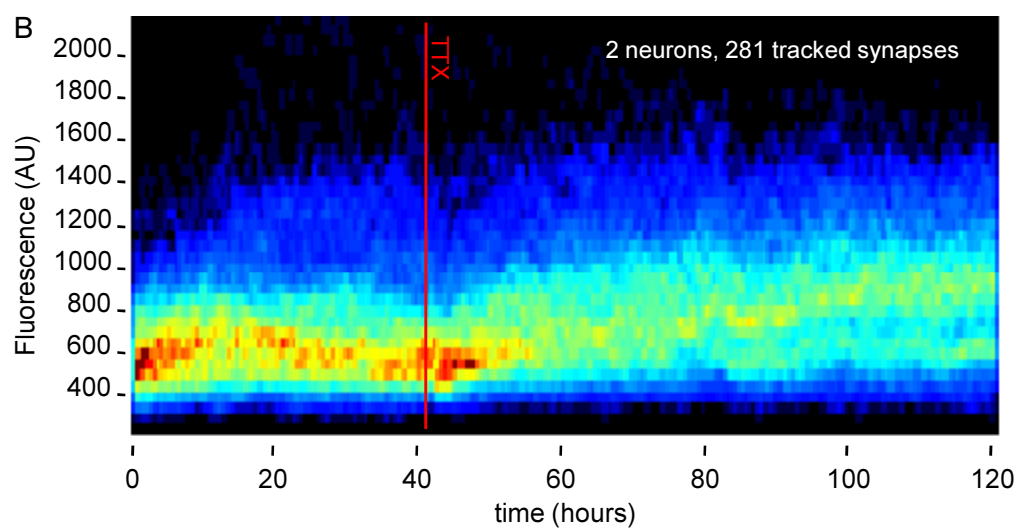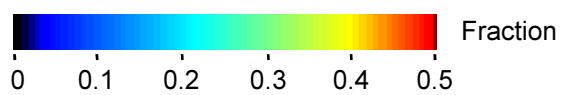

Supplement: Figure S5 — Comparison of fluorescence intensity distributions for all PSD-95:GFP puncta and tracked puncta. (A) Normalized distribution of fluorescence intensities of all discernable PSD-95:GFP puncta at each time point (same data as Figure 7E). (B) Normalized distribution of fluorescence intensities of all 281 tracked puncta in this experiment. (0.03 MB PDF) [file pbio.1000136.s005.pdf]

A

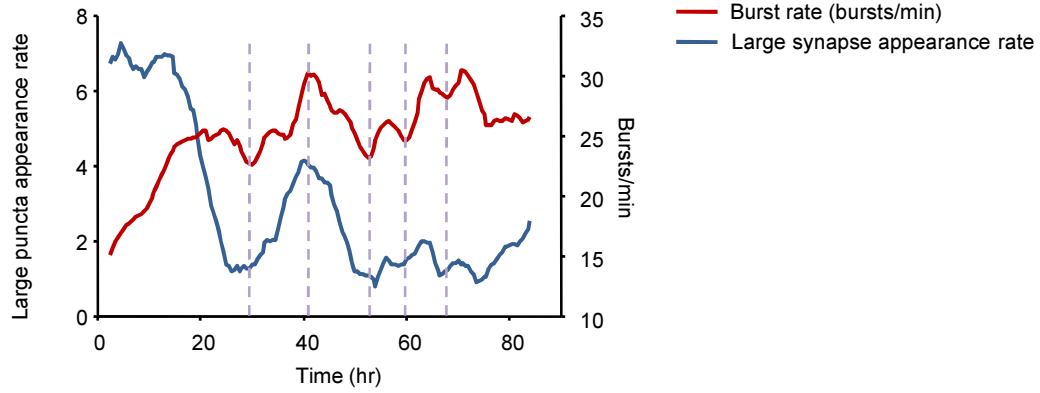

B

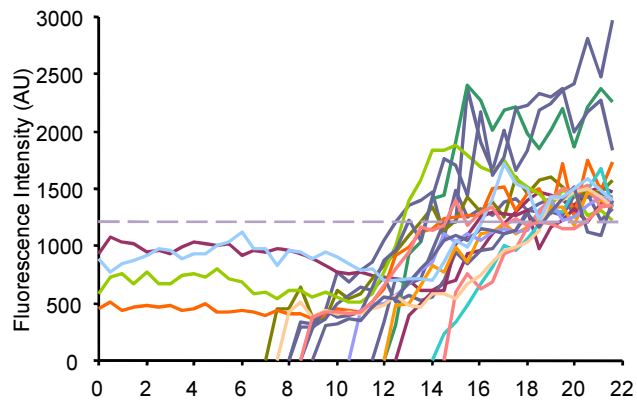

C

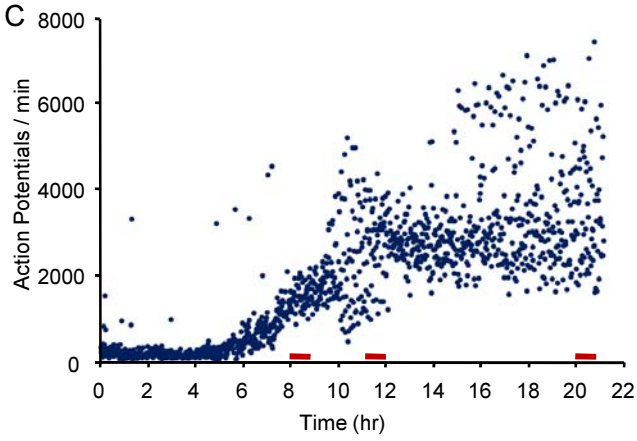

D

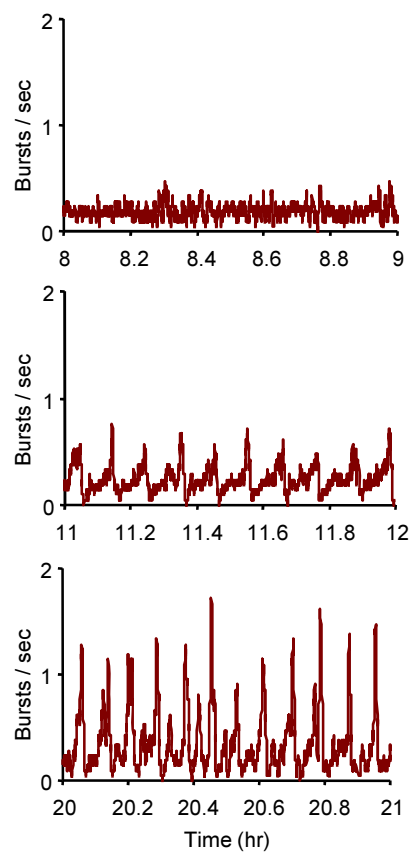

Supplement: Figure S6 — Synchronous activity drives the appearance of particularly large synapses. (A) Temporal correlations between burst rates and the appearance rates of bright synapses. Bright puncta were examined in a sliding time window of 5 h. A global threshold was defined (1.5 standard deviations above mean PSD-95:GFP puncta fluorescence). Puncta were counted if their brightness was at least 200 fluorescence units below the threshold at the beginning of the time window and exceeded the threshold at the end of the time window. Burst counts were smoothed with a 2-h kernel. Same experiment as that of Figure 4. (B) Eighteen bright PSD-95:GFP puncta at t = 22 h were tracked backwards in time to the beginning of the experiment. Each line denotes the fluorescence intensity of one punctum. Horizontal dashed line indicates mean+1.5 standard deviations. Same experiment shown in Figure 6. (C) Network activity levels during the same period. (D) Bursts rates for three 1-h periods in this experiment (red bars in [C]). Data were smoothed using a 60-s kernel. (0.12 MB PDF) [file pbio.1000136.s006.pdf]
