# Supplementary material for: Long-Term Relationships between Synaptic Tenacity, Synaptic Remodeling, and Network Activity
Source: PLoS Biol. 2009 Jun 23;7(6):e1000136. doi: 10.1371/journal.pbio.1000136 (PMC2693930; doi:10.1371/journal.pbio.1000136)
Supplement: Figure S7 — Relationships between changes in fluorescence in consecutive time windows, before and after addition of TTX. Absolute changes in fluorescence (two top rows) and fractional changes (two bottom rows) in consecutive 10-h time windows straddling the moment of TTX application. Data are shown in raw form (first and third rows) and in normalized form: after correcting for the mean change of the entire population in each time window (second and fourth rows). This analysis indicates that most PSDs that had recently experienced considerable growth or shrinkage do not seem to be particularly protected from subsequent change upon complete activity blockade. (1.05 MB PDF) [file pbio.1000136.s007.pdf]

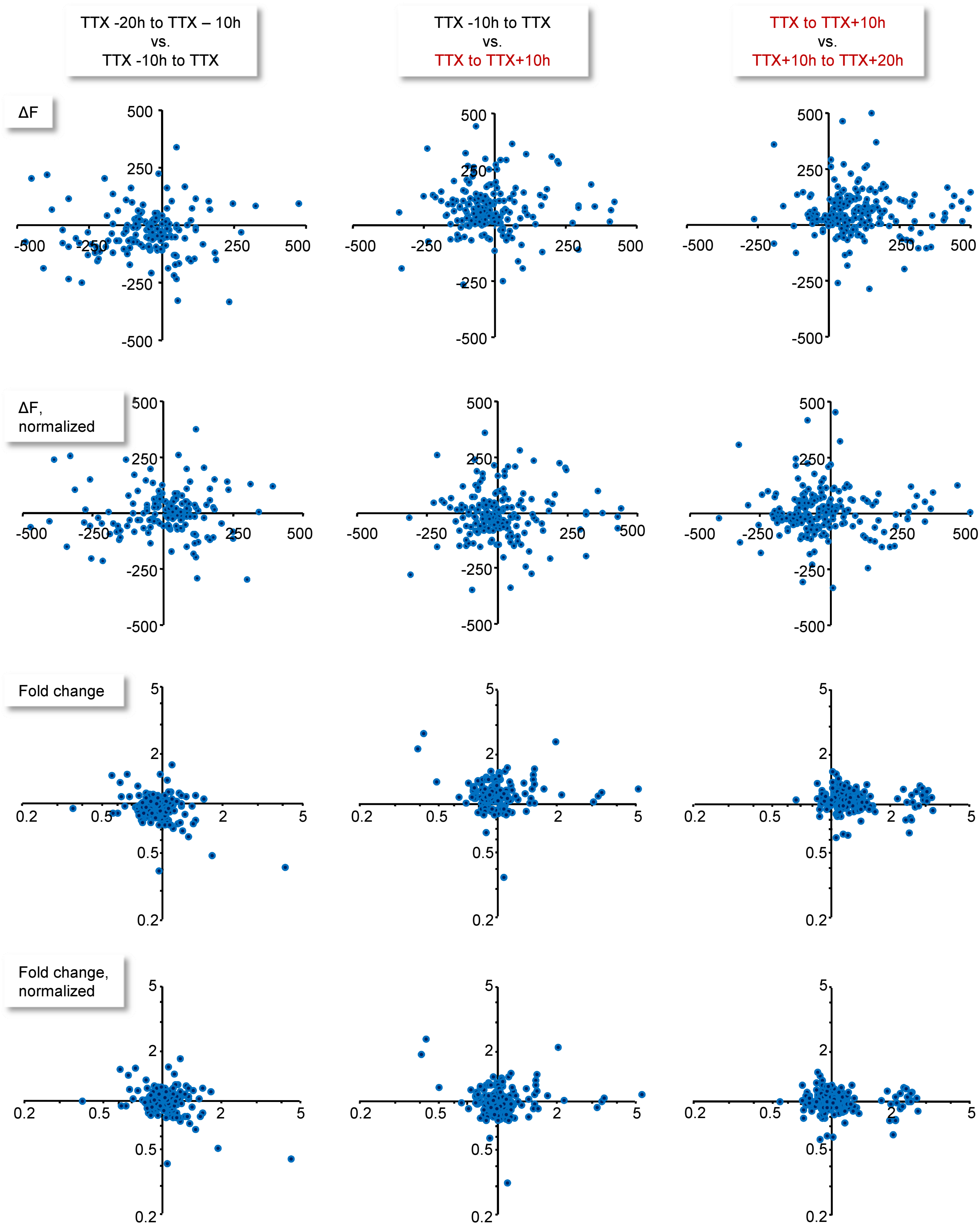

TTX -20h to TTX – 10h
vs.
TTX -10h to TTX
TTX -10h to TTX
vs.
TTX to TTX+10h
TTX to TTX+10h
vs.
TTX+10h to TTX+20h
ΔF
ΔF,
normalized
Fold change
Fold change,
normalized
500
250
-250
-500
-500
-250
250
500
5
2
0.5
0.2
0.2
0.5
2
5
